# A large-scale multimodal investigation of the interplay between the serotonergic system and emotion processing

**DOI:** 10.1038/s41398-025-03407-2

**Published:** 2025-06-11

**Authors:** Manfred Klöbl, Matej Murgaš, Murray Bruce Reed, Leo Robert Silberbauer, Annette M. Hartmann, Godber Mathis Godbersen, Gregor Gryglewski, Lukas Nics, Andreas Hahn, Dan Rujescu, Marcus Hacker, Rupert Lanzenberger

**Affiliations:** 1 Department of Psychiatry and Psychotherapy, Medical University of Vienna, Vienna, Austria; 2 Comprehensive Center for Clinical Neurosciences and Mental Health, Medical University of Vienna, Vienna, Austria; 3 Child Study Center, Yale University, New Haven, CT USA; 4 Department of Biomedical Imaging and Image-guided Therapy, Division of Nuclear Medicine, Medical University of Vienna, Vienna, Austria

**Keywords:** Depression, Molecular neuroscience

## Abstract

Considering the complexity of serotonergic influence on emotions, we conducted a comprehensive investigation of the interplay between emotion processing and the serotonergic system using simultaneous functional and molecular neuroimaging during pharmacological challenge while disentangling the effects of serotonin transporter (SERT) binding, genotype, and diagnosis of major depressive disorder (MDD). Herein, 153 subjects (44 with MDD) performed a facial emotion processing task during functional magnetic resonance imaging (fMRI) before and after an acute intravenous application of 8 mg citalopram or placebo. Patients with MDD were assessed again after at least three months of antidepressant treatment. Citalopram administration resulted in a reduced fMRI activation in regions involved in fear processing, including the anterior cingulate cortex (ACC), when viewing fearful faces contrasted against happy or neutral faces. ACC activation correlated negatively with striatal/thalamic SERT availability across drug conditions as measured by [11 C]DASB positron emission tomography. Across groups, citalopram-induced changes in ACC activation correlated with emotional attribution, indicating stronger reductions for subjects with higher self- versus other- attribution. Moreover, striatal SERT availability mediated the influence of the number of 5-HTTLPR/rs25531 L_A_ alleles on ACC activation under placebo. Patients with MDD exhibited increased activations in the intraparietal and superior frontal sulcus in response to fearful versus happy faces at baseline, and along the parieto-occipital/calcarine fissure after treatment. We interpret our findings on multiple levels of the serotonergic-emotional interaction within the context of enhanced passive coping and acute anxiolytic effects of citalopram following potential changes in serotonin or SERT availability.

## Introduction

Understanding the interplay between the serotonergic system and emotion processing is crucial for elucidating the neurobiology of affective disorders such as major depressive disorder (MDD). The modulatory neurotransmitter, serotonin, is centrally involved in regulating mood and emotion. By blocking the serotonin transporter (SERT), selective serotonin reuptake inhibitors (SSRIs) increase the availability of free serotonin in the synaptic cleft. While SSRIs remain the standard treatment for major depressive disorder (MDD), they affect emotion processing in patients as well as healthy individuals [1–3].

Functional magnetic resonance imaging (fMRI) studies frequently use facial expressions to probe the neural correlates of emotion processing. Such investigations include the interaction with the serotonergic system [4] and valence-dependent effects of SSRIs in healthy individuals and patients with MDD [5–7]. Experiments combining fMRI with positron emission tomography (PET) shed further light on the effect of the serotonergic system on brain activity during emotion processing [8, 9].

The SLC6A4 gene encoding SERT has numerous variants, the length polymorphism 5-HTTLPR being the most widely studied with L_A_/L_A_ (homozygotes for the long allele and adenine at SNP rs25531) showing the highest average SERT expression in previous studies [10–12]. The genotypic groups were associated with structural and functional variations in the anterior cingulate cortex (ACC) and the amygdala, as well as the connectivity between the regions [13–16]. Moreover, imaging genetics investigating effects of SERT-linked promoter region (5-HTTLPR) polymorphisms on emotion processing suggested that activation in multiple parts of the limbic system is affected by SERT expression in a complex manner (see [4, 17] for comprehensive reviews). However, SERT expression and emotion processing-related brain activation are influenced by a multitude of known and unknown factors and the exact role of the 5-HTTLPR polymorphism is not entirely understood. For instance, SERT binding but not the 5-HTTLPR genotype were found to be related to brain activation in response to negative emotional stimuli in MDD [18]. Moreover, even though the 5-HTTLPR genotype is often mentioned as influential on SERT availability, such a relationship is not always found even in larger studies [19, 20].

Altogether, previous work indicated a complex and intricate relationship between MDD-, emotional valence-, SSRI application, and gene expression-dependent effects on emotion processing. Integrating these aspects, the present study aims to extend our understanding of serotonin’s role in emotion processing by adopting a comprehensive approach from various perspectives. These include i) fMRI of emotional stimuli, ii) molecular imaging of the SERT binding, iii) a pharmacological challenge and treatment with an SSRI, iv) SERT genotyping, and v) psychometric scales in both healthy control subjects (HCs) as well as patients with MDD. Specifically, we implement an explicit emotion identification task that is performed before and after receiving an intravenous citalopram challenge or placebo in a double-blind crossover design.

During task performance, we simultaneously acquired fMRI/PET data to assess brain activation and to quantify SERT availability and citalopram binding using the radioligand [^11^C]-N,N-dimethyl-2-(2-amino-4-cyanophenylthio)-benzylamine ([^11^C]DASB). 5-HTTLPR allele length and SNP rs25531 genotyping provided further information on SERT expression. To allow for contrasting acute and chronic effects, patients with MDD underwent a follow-up MRI measurement while receiving pharmacotherapy. Finally, we integrated depressive symptom severity and attribution styles (i.e., characteristic ways in which individuals interpret and evaluate the causes of events, behaviors, or outcomes, whether they are positive or negative) as psychometric measures of emotion processing into our analyses.

Linking our data on various aspects of the serotonergic system, we first examined whether a diagnosis of MDD, a psychiatric condition with known serotonergic system involvement, affects brain activation during emotion processing. This was assessed before and after three months of antidepressant therapy to evaluate the effects of chronic serotonergic drug application on emotion processing. Given that emotion processing is influenced by serotonergic signaling, we then investigated how an acute challenge with the SSRI citalopram modulated related brain activation in both healthy individuals and patients with MDD. To establish a connection between serotonergic modulation of emotion processing and the underlying SERT distribution, brain activation in affected regions was subsequently related to the [^11^C]DASB binding under placebo and citalopram, which provide indices of SERT availability and drug occupation. We then explored a potential pathway from gene expression over transporter expression to neural activation by testing whether the influence of the 5-HTTLPR polymorphism on brain activation is modulated by the previously established link to SERT binding. Our final psychometric perspective on the interplay between the serotonergic system and emotion processing comprised correlation analyses of fMRI brain activation with emotional attribution and depressive symptoms.

## Materials and methods

### Study design

**Fig. 1:**
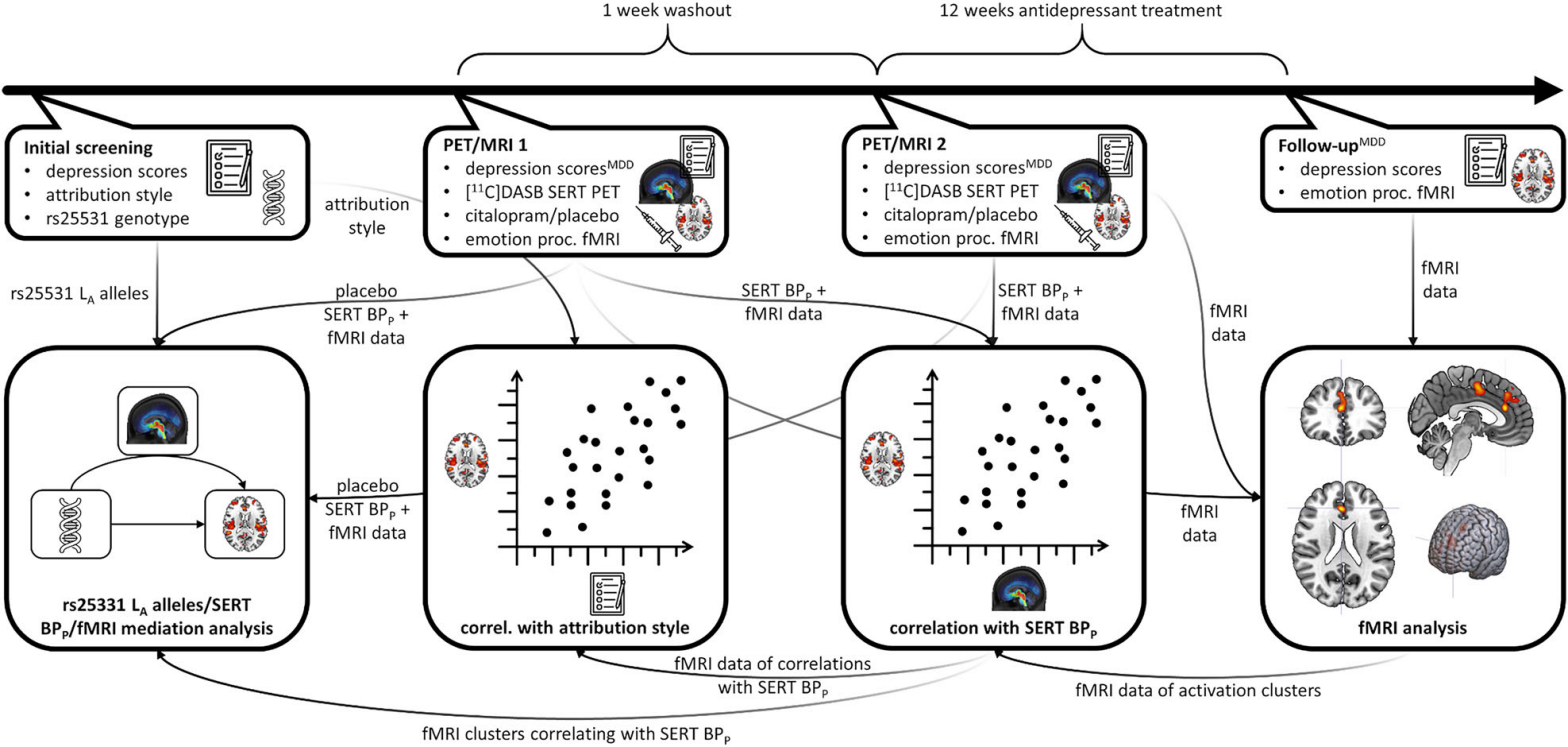
Study procedure and analysis workflow. The steps marked with ^MDD^ were conducted in the group of patients with major depressive disorder (MDD) only. PET: positron emission tomography, MRI: magnetic resonance imaging, [^11^C]DASB: [^11^C]-N,N-dimethyl-2-(2-amino-4-cyanophenylthio)-benzylamine, SERT: serotonin transporter, fMRI: functional MRI, BP_P_: specific binding potential, L_A_: long allele with adenine, IPSAQ-R: Internal, Personal and Situational Attributions Questionnaire – revised.

The study followed a randomized, double-blind, placebo-controlled crossover design in HCs and patients with MDD. Patients with MDD underwent a subsequent open-label treatment phase. The crossover stage of the study consisted of two PET/MRI assessments with double-blind, placebo-controlled infusion of 8 mg citalopram. All subjects performed one run of an emotion identification task before and one after intravenous administration of the study medication [21]. The follow-up MRI assessment in the MDD group took place at least three months after initiation of the open-label antidepressant treatment. After scanning, treatment was initiated with 10 mg escitalopram. Dosage was adapted according to clinical response defined as a minimum reduction of 50% in the Hamilton Depression Rating Scale (HAM-D) and the drug switched only if necessary. The current analyses also included additional fMRI and PET data of healthy individuals participating in a preceding pilot study with similar design (see [22, 23] for preliminary analyses of the pilot data). We conducted the pilot study on healthy subjects to verify the emotion identification task’s sensitivity to citalopram. Genetic and psychometric data, as described below, was collected during the main study only. See Fig. 1 for an overview of the study procedure and analyses conducted.

The study was conducted according to the Declaration of Helsinki including revisions, the good clinical and scientific practice guidelines of the Medical University of Vienna, and approved by the local ethics committee (number 1307/2014). Written informed consent was obtained from all subjects upon inclusion. This study was preregistered at clinicaltrials.gov (NCT02711215).

### Subjects

In total, we enrolled 204 subjects (50 with MDD) with 153 subjects (44 with MDD) providing 561 runs of the emotion identification task. Partially available data was included whenever possible. The Structured Clinical Interview for DSM-IV (SCID-I) confirmed a diagnosis of MDD in the patient cohort or ruled out comorbidities as well as any mental disorders in HCs. Patients with MDD also required a HAM-D score of ≥ 18 to be included. For details on the inclusion and exclusion criteria, please refer to the supplement. Subjects were block-randomized to a sequence of drug application (placebo or citalopram first) in matched pairs across groups (MDD, HC) and stratified for sex.

### Emotion identification task

The task is an adaptation to the established facial emotion discrimination task [24, 25] showing single faces for differentiation of emotions. Explicit over implicit emotion processing should ensure attention in spite of the long scanning sessions.

Subjects saw pictures of single individual faces with happy, fearful or neutral expressions [26, 27]. The control condition comprised scrambled versions of the face pictures preserving the approximate color distribution. For each image, subjects were required to signal whether it depicted a negative, neutral or positive facial expression or if they could not clearly recognize the expression using an MR-compatible keyboard. The images were displayed for a duration of 3 s and were separated by uniformly distributed baseline periods of 3-7 s showing a fixation cross. Each subject viewed 17 pictures of each category in a randomized order. The pre- and post-drug application runs contained different faces to reduce habituation. The pilot version of the task did not include the scrambled pictures but 20 stimuli of each category and had baseline periods of 3.5-8.5 s.

### Functional magnetic resonance imaging acquisition and analysis

The fMRI data was acquired on a Siemens Biograph mMR 3 T PET/MRI scanner (Siemens, Erlangen, Germany) with the following parameters: TE/TR = 30/2440 ms, voxel size = 2.1 × 2.1 × 3.0 mm, gap = 0.75 mm, 100 × 100 voxels in-plane, 36 slices, GRAPPA 2 [28, 29]. Subject-level models included one regressor per emotion/control condition. The contrasts of the conditions against each other were carried forward to the group-level analysis. For the detailed processing steps, please refer to the supplement.

### Whole-brain fMRI analysis

Group-level analyses using the Sandwich Estimator toolbox, version 2.2.2 allowed for the inclusion of subjects with only partially available data [30]. The analysis included group (HC, MDD), condition (placebo, verum, follow-up), and run (pre-, post-drug application) as regressors of interest, along with their second- and third-order interaction terms (follow-up scans were not included in interactions as only patients with MDD participated in them). The model was adjusted for subject sex and age, study phase (pilot or main study), order of drug conditions, and session effects (excluding the last two in follow-up analyses due to collinearity). AFNI’s 3fFWHMx and 3dClustSim were employed for multiplicity correction on cluster-level to p < 0.05, corrected, with a primary threshold of p < 0.001, uncorrected, both two-sided. Additional Šidak correction adjusted for the number of investigated contrasts. Median contrast values of significant clusters were extracted for visualization and to run linear mixed effects (LME) models checking for two-sided significant baseline differences, followed by multimodal analyses.

### [^11^C]DASB positron emission tomography acquisition and processing

PET data was acquired in parallel to the fMRI data on the same scanner. [^11^C]DASB was synthesized in-house at the Department of Biomedical Imaging and Image-guided Therapy, Division of Nuclear Medicine, Medical University of Vienna [31]. The tracer was applied as a 1-min bolus followed by constant infusion through the cubital vein for 180 min. PET acquisition started 35–45 min after the initial bolus and lasted for 120 min. The PET data was corrected for the decay, scatter, random coincidences, detector dead time, and attenuation using low-dose computed tomography scans of the subjects’ heads (Siemens Biograph TruePoint PET/CT). Afterwards, PET data was reconstructed to 24 frames (5 × 2 min, 16 × 5 min, 3 × 10 min). The pharmacological challenge (citalopram or placebo) was applied 70 min after the bolus application. Arterial blood samples were collected from the radial artery at 40, 60, 65, 69, 72, 75, 78, 82, 88, 100, 120, 130, and 140 min after bolus application. The blood samples were used to measure the whole-blood and plasma activity, as well as the fraction of unmetabolized parent compound in plasma, which served to calculate the arterial input function at each time point. Specific binding potential (BP_P_) was calculated as the ratio between the tissue activity and the arterial input function acquired between 120 and 140 min after bolus application using the equilibrium method [32]. BP_P_ and SERT occupancy for citalopram versus placebo were extracted from thalamus and striatum due to their high signal-to-noise ratio and implications in MDD using an in-house atlas [33–35].

### 5-HTTLPR/rs25531 genotyping

Genotyping of the 5-HTTLPR variant was performed using polymerase chain reaction followed by gel electrophoresis as previously described [36]. Five genotype groups were derived from the 5-HTTLPR length polymorphism and SNP rs25531: L_A_/L_A_, L_A_/L_G_, L_A_/S, L_G_/S, S/S [11]. For details, please refer to the supplement.

### Psychometric assessment and evaluation

We assessed depressive symptom severity using the HAM-D, Montgomery-Åsberg depression rating scales (MADRS) and Beck’s depression inventory (BDI). The questionnaires were administered in both groups at the screening visit. In addition, the MDD group completed the questionnaires at both MRI sessions as well as during five subsequent visits at approximately two-week intervals and at the follow-up MRI session. Furthermore, we assessed individual attribution styles at the screening visit using the Internal, Personal and Situational Attributions Questionnaire – revised (IPSAQ-R [37]) since these might provide a richer psychometric description of how especially HCs perceive and attribute emotions rather than a lack of depressive symptoms.

The first two principal components of the six IPSAQ-R scales were extracted. Only the first principal component, comprising the difference between self- (positive weight) and other-attribution (negative weight) for both positive and negative events, showed relevance in later analyses. For missing values and imputation details, see supplement.

### Multimodal analysis

Using LMEs, we analyzed how variations in SERT BP_P_ and citalopram-induced occupancy affected the clusters of significantly changing fMRI brain activation under different drug conditions. In addition to striatum/thalamus BP_P_ or occupancy, the models contained regressors for group, condition, sex, age, study phase, session, order effects and a random intercept per subject, as applicable.

For BP_P_ correlations, activations were either taken from the difference of post- and pre-drug application runs (less biased by subjects’ daily condition) or the post-application runs alone (less variance). Concerning occupancy correlations, we calculated the citalopram-placebo fMRI activation differences.

If fMRI activation clusters showed significant correlations with BP_P_ or occupancy, subsequent analyses involved correlating their citalopram-placebo fMRI activation differences with scores from the IPSAQ-R to investigate associations with attribution style. Furthermore, exploratory analyses were conducted to determine whether significant differences found for follow-up scans were related to changes in depressive symptoms, given the limited number of returning patients with MDD.

Given the strong correlations between the potential regressors and to reduce the number of comparisons, the first principal components of striatum and thalamus was used. The sine-similarity approach was used to correct p-values (indicated with p_SiSi_) for the number of correlations [38].

Lastly, investigations were carried out to test whether significant correlations of SERT availability in striatum/thalamus with emotion identification task activation mediated the influence of the number of L_A_ alleles of the 5-HTTLPR/rs25531 polymorphism (R package “mediation” [39]; see supplement for details). Since several previous works describing significant differences in SERT availability depending on the 5-HTTLPR genotype did not find this effect in the thalamus, we only used the striatal BP_P_ for the mediation analysis [11, 40, 41]. Furthermore, we conditioned the test on age to mitigate known age-effects on SERT expression [42]. Lastly, we standardized the unitless fMRI activation and BP_P_ in this model to express the influence of the number of L_A_ alleles in terms of standard deviations. Model diagnostics were visually assessed. Two-sided p-values are reported for all multimodal analyses.

## Results

Demographic details, distributions of the 5-HTTLPR polymorphism, and psychometrics are presented in Table 1.

**Table 1:** Overview of demographics, serotonin transporter-linked promoter region polymorphism distribution, serotonin transporter (SERT) occupancy by citalopram, and psychometrics.

	HC pilot	HC main	MDD main
N (female, complete)	fMRI: 15 (6, 11) PET: 14 (5, 13)	fMRI: 94 (55, 68) PET: 68 (37, 52) genotyping: 128 (74, -) psychometrics: 81 (50, -)	fMRI: 44 (21, 35) PET: 41 (20, 34) genotyping: 50 (24, -) psychometrics: 47 (21, -) follow-up fMRI: 29 (13, -)
Age (any data)	23.44 ± 4.95	25.05 ± 9.50	26.35 ± 8.48
L_A_/L_A_ carriers		23	16
L_A_/L_G_ carriers		9	1
L_A_/S carriers		61	21
L_G_/S carriers		6	2
S/S carriers		29	10
Thalamic SERT occupancy [%]	70.36 ± 20.56	69.80 ± 8.63	67.08 ± 8.95
Striatal SERT occupancy [%]	67.36 ± 21.15	65.02 ± 8.69	65.61 ± 6.32
HAM-D		baseline: 0 ± 0	baseline: 19.67 ± 3.33 follow-up: 5.00 ± 4.50
MADRS		baseline: 0 ± 0	baseline: 27.50 ± 6.58 follow-up: 8.00 ± 13.00
BDI		baseline: 0 ± 0	baseline: 25.33 ± 10.63 follow-up: 9.50 ± 15.00
IPSAQ-R positive self [%]		50.53 ± 17.50	52.16 ± 14.63
IPSAQ-R positive other [%]		34.63 ± 14.44	35.26 ± 9.92
IPSAQ-R positive situation [%]		11.66 ± 13.89	9.49 ± 10.39
IPSAQ-R negative self [%]		40.63 ± 20.38	49.38 ± 16.36
IPSAQ-R negative other [%]		38.13 ± 19.88	39.87 ± 9.06
IPSAQ-R negative situation [%]		16.88 ± 16.68	10.63 ± 9.85

Summary statistics are median ± interquartile range.

*fMRI* functional magnetic resonance imaging, *PET* positron emission tomography, *L* long, *S* short, *A* adenine, *G* guanine, *HAM-D* Hamilton depression rating scale, *MADRS* Montgomery-Åsberg depression rating scales, *BDI* Beck’s depression inventory, *IPSAQ-R* Internal, Personal and Situational Attributions Questionnaire-revised.

### Emotion identification task – depression-related effects

We found significant baseline group differences across drug conditions at the intraparietal/postcentral and superior frontal sulcus with higher activation in the MDD than the HC group for fearful versus scrambled faces. These findings were not present when comparing the HC with the MDD group at follow-up. After chronic antidepressant treatment, we found increased activation for fearful versus happy faces in the MDD group along the crossing of the left calcarine and parieto-occipital fissure. We found no significant correlations between the differences in follow-up and baseline depression scores and activation for fearful versus happy faces in the cluster along the parieto-occipital/calcarine fissure (p > 0.16 for all depression scores). Of note, six patients with MDD were switched to combined serotonergic/noradrenergic drugs during the follow-up period (supplement).

### Emotion identification task – citalopram-related effects

**Fig. 2:**
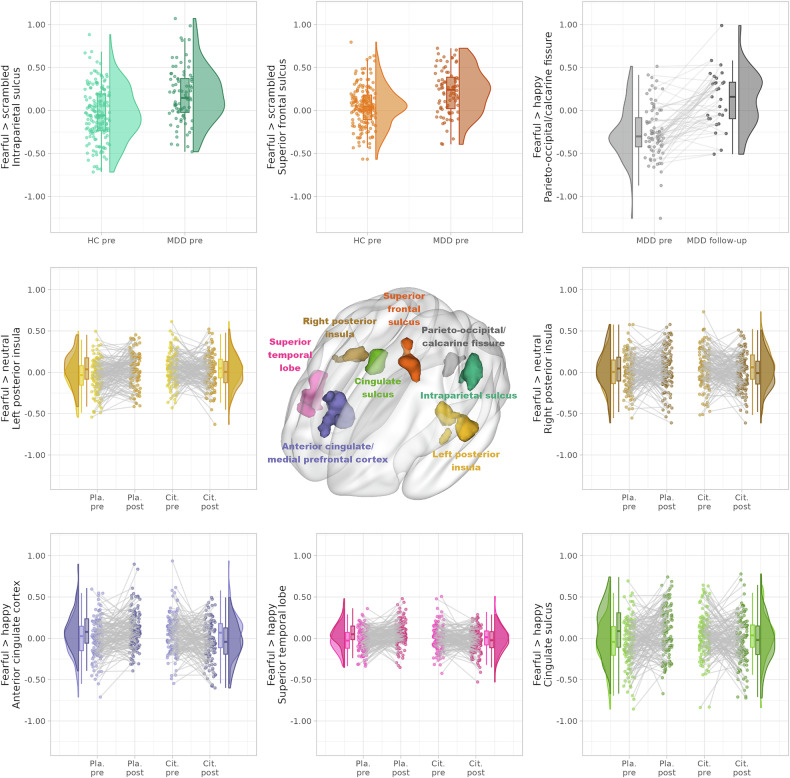
Significant results of the whole-brain and multimodal analyses. The top row shows the regions with significantly higher activation in patients with major depressive disorder (MDD) compared to healthy controls (HCs) before application of the study drug (pre) for the contrast of fearful versus scrambled faces as control stimuli, i.e., at baseline. Furthermore, the MDD group showed significantly increased activation for fearful versus happy faces after at least three months of open-label antidepressant treatment (follow-up). The second and third row show significant interaction effects before (pre) compared to after (post) intravenous application of either citalopram (Cit.) or placebo (Pla.) with relative decreases in activation after citalopram for fearful versus happy or neutral faces.

**Fig. 3:**
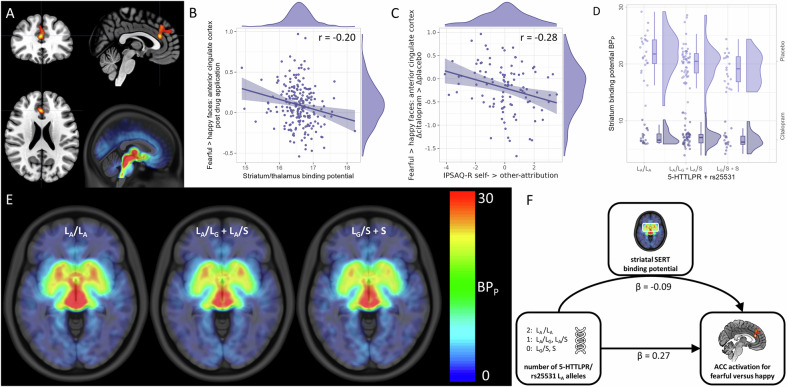
Correlation and mediation analyses. **A** Cluster in the anterior cingulate and medial prefrontal cortex (ACC) showing reduced activation for fearful versus happy faces under citalopram. Distribution of serotonin transporter binding potential (SERT BP_P_). **B** The ACC activation negatively correlates with the BP_P_ in striatum and thalamus across placebo and citalopram application. **C** The difference between citalopram and placebo in activation of the ACC cluster negatively correlates with self- versus other-attribution in the Internal, Personal and Situational Attributions Questionnaire - revised (IPSAQ-R). **D** Striatal BP_P_ correlates with the number of L_A_ alleles of the 5-HTTLPR/rs25331 (SERT-linked promoter region) polymorphism under placebo. **E** Axial slice though the striatum showing the BP_P_ under placebo dependent on the number of L_A_ alleles. **F** Mediation analysis showing that the number of L_A_ alleles exerts a negative influence on ACC activation for fearful versus happy faces via striatal SERT BP_P_. The number of L_A_ alleles also shows a direct positive influence on ACC activation. Color maps in A and E were chosen for good visibility only with warmer colors indicating stronger effects.

Significantly reduced activations under citalopram were identified in cingulate, frontal and temporal regions for fearful versus happy faces (we refer to the cluster with peak in the ACC and stretching into the medial prefrontal cortex as ACC for the sake of simplicity, see Fig. 2, Fig. 3, Table 2). The bilateral posterior insula further showed significantly reduced activation under citalopram for fearful versus neutral faces. There was a significant baseline difference in pre-drug application runs only in the activation of the left posterior insula (with reduced activation only before placebo application, p = 0.0029, LME on extracted values, uncorrected). The MDD group did not react significantly different to citalopram versus placebo from the HCs on the whole-brain level, i.e., there was no significant group-by-substance-by-run interaction and the above effects were observed across both groups. See Table 2 and Fig. 2 for the detailed whole-brain results (base contrasts in supplement).

**Table 2:** Whole brain results for the facial emotion identification task.

Comparison	Contrast	Cluster size [voxel]	Peak coordinates			p-value corrected	p-value cluster-level	Region
			x	y	z			
MDD > HC	fearful > scrambled	283	−52	−34	50	0.0025	0.0005	intraparietal/ postcentral sulcus
		123	−44	30	32	0.0961	0.0200	middle frontal gyrus
		154	−28	−2	60	0.0369	0.0075	superior frontal sulcus
		105	18	−14	68	0.1610	0.0345	central sulcus
citalopram < placebo	fearful > happy	256	2	32	22	0.0025	0.0005	ACC, superior medial frontal gyrus
		200	62	0	−14	0.0100	0.0020	superior temporal gyrus + sulcus
		174	4	−4	50	0.0174	0.0035	cingulate sulcus
		142	48	−16	8	0.0514	0.0105	superior temporal gyrus
	fearful > neutral	373	−38	−20	14	0.0025	0.0005	posterior insula
		108	−10	10	36	0.1457	0.0310	cingulate sulcus
		159	42	−30	14	0.0321	0.0065	posterior insula
	fearful	102	52	8	8	0.1782	0.0385	anterior insula, Rolandic operculum
		100	−60	4	22	0.1910	0.0415	central sulcus
follow-up > MDD baseline	fearful > happy	148	−8	−66	14	0.0442	0.0090	parieto-occipital sulcus, calcarine gyrus
follow-up > MDD citalopram	fearful > happy	125	4	46	32	0.0891	0.0185	superior frontal gyrus

The “MDD > HC” comparisons were conducted with the pre-drug application runs only. “Citalopram < placebo” describes the interaction effect of drug condition and pre-/post-drug application run over both subject groups. The follow-up comparisons include the pre-drug application runs of the patients with major depressive disorder (“MDD baseline”) and after intravenous application of 8 mg citalopram (“MDD citalopram”). All results are corrected at cluster-level with a primary threshold of p < 0.001 and family-wise error (FEW) threshold of p < 0.05, both two-sided. An additional correction was performed for the five contrasts compared. Approximate regions are based on the atlas of intrinsic connectivity of homotopic areas (AICHA [77]).

### Relation between brain activation and serotonin transporter binding

A significant negative correlation between striatum/thalamus SERT BP_P_ and ACC activation for fearful versus happy faces across both drug conditions and both subjects groups for the post-application runs was found (r = −0.20, p_SiSi_ = 0.0306; Fig. 3). We found no significant correlations between fMRI task activation and SERT occupancy.

Visual inspection revealed a grouping of striatal BP_P_ under placebo according to number of L_A_ alleles (Fig. 3). Mediation analysis indicated a significant negative influence of the number of L_A_ alleles via striatal SERT BP_P_ on ACC activation for fearful versus happy faces (β = −0.09 standard deviations of ACC activation per L_A_ allele, p = 0.0430). The direct effect of the number of L_A_ alleles on ACC activation was positive and of higher magnitude but not significant (β = 0.27, p = 0.0950). The direct effect became significant when averaging over all non-citalopram runs (β = 0.40, p = 0.0060; see supplement).

### Relation between brain activation and attribution styles

The first principal component of the IPSAQ-R (i.e., self- versus other-attribution, with higher values indicating more self- and less other-attribution) showed a significant negative correlation with the citalopram-placebo difference for fearful versus happy ACC activation corrected for the pre-drug application runs (r = −0.28, p_SiSi_ = 0.0191; Fig. 3). Follow-up analyses revealed similar but opposing correlations for self- (r = −0.26) and other-attribution (r = 0.27) separately.

## Discussion

Employing an explicit emotion processing task, decreased activation under acute intravenous citalopram application compared to placebo when viewing fearful versus happy or neutral faces were identified. In addition, higher activations were identified in patients with MDD compared to HCs at baseline and increases within the MDD group after compared to before chronic antidepressant treatment. Activation in the ACC for fearful versus happy faces negatively correlated with striatal/thalamic SERT availability. Under placebo, striatal SERT availability mediated the influence of the 5-HTTLPR/rs25531 polymorphism L_A_ allele count on ACC activation for fearful versus happy faces. Moreover, the change in ACC activation after receiving citalopram versus placebo negatively correlated with the difference of self- and other-situational attribution of events.

### A potential relation between the anterior cingulate cortex, SERT binding, and passive coping

Modulatory influences of the 5-HTTLPR/rs25531 polymorphism and serotonergic agents on emotion-related activation in anterior cingulate/prefrontal regions are well documented [4, 17]. Previous studies mainly point towards increased activation in the ACC in response to emotional face stimuli in S allele carriers with a decrease observed under antidepressant therapy [4]. Moreover, it has been reported that the highest SERT expression occurs in homozygotic L_A_ carriers [10, 11]. While focusing on the number of L_A_ alleles, our findings align with the previous literature showing reductions in activation induced by citalopram, and additionally indicate that the number of 5-HTTLPR L_A_ alleles influences ACC activation via SERT availability.

While ACC and medial prefrontal cortex are involved in fear processing [43–45] and respond to emotion induction in general [46], a recent study hypothesized that serotonergic modulation affects the variability in emotional responses, in particular of the excitability and connectivity of ACC and amygdala [9]. This variability might underlie the fact that the contrasts in our observed interaction effects per condition are on average often close to zero (e.g., for the ACC, Fig. 2). Thus, citalopram might rather influence the difference in neural responses between emotions than for a single emotion. This interpretation is tempting given it would provide a fast neural signature of the decrease in emotional responsiveness frequently observed during SSRI treatment [47] and their proposed effect of increased passive coping [48].

SSRIs might strengthen passive coping capabilities via indirect effects on the 5-HT_1A_ receptor rather than directly at the SERT [48]. Links between 5-HT_1A_ binding and 5-HTTLPR [49] as well as SERT density [50] could imply an indirect association between SERT expression and passive coping. In particular, the involvement of the medial prefrontal cortex and the amygdalae in coping behavior might be influenced via 5-HT_1A_ receptors of serotonergic dorsal raphe neurons [51]. While the delayed antidepressant and anxiolytic effects of serotonergic agents are presumably related to an increased activation of postsynaptic 5-HT_1A_ heteroreceptors following the desensitization of presynaptic 5-HT_1A_ autoreceptors [52, 53], there is also meta-analytic evidence form animal studies for acute anxiolytic effects of citalopram and other SSRIs [54, 55]. SSRIs reduced conditioned fear with some advantages of chronic as compared to subchronic or acute treatment [54]. The strength of the anxiolytic effect was independent of the treatment duration for unconditioned fear [55]. However, the results of the underlying animal studies are heterogeneous, with some reporting anxiolytic effects, some no effects, and some even anxiogenic effects of SSRIs. The meta-analyses also indicated a publication bias and thus overestimated effects. Still, the reduced activation to fearful faces we observed might constitute neural correlates of acute anxiolytic effects. Hence, there might be effects of citalopram fostering passive coping before chronic administration – at least with regard to fear.

The negative correlation between SERT BP_P_ and fear-related ACC activation on the one hand, and the decrease in this activation under acute citalopram could be reconciled in two potential ways: Firstly, despite that SERT is taking up serotonin, it does not follow that higher SERT binding correlates with lower extracellular serotonin concentrations. Contrarily, higher SERT binding might be reflective of upregulation in the presence of higher serotonin concentrations [56, 57]. Considering earlier work in rats showing that presynaptic serotonin reduced excitatory signaling in the cingulate cortex [58], the negative correlation between SERT BP_P_ and ACC activation might be explained by SERT binding being reflective of baseline serotonin concentrations, and therefore individuals with higher SERT binding exhibiting attenuated ACC activations. Consequently, the reduction in ACC activation observed after acute SSRI application might be indicative of an increase of serotonin concentrations.

Secondly, both higher SERT BP_P_, i.e., SERT density, and an initial increase in 5-HT_1A_ autoreceptor activation in the raphe nuclei following citalopram administration [59] might lead to decreased levels of serotonin available in the synaptic cleft [60, 61]. This perspective is backed up by findings of reduced serotonin concentrations in serotonergic projection areas in human and non-human primates [62], which might correspond to reduced excitatory signaling in the cingulate cortex as found in rats [58]. A more recent finding in rats linked the raphe-ACC signaling pathway to restored social behavior [63]. Thus, an initial decrease of serotonin levels in the synaptic cleft though 5-HT_1A_ autoreceptor activation, might play a role in the decrease of ACC activation particularly when viewing fearful faces. The plausibility of both potential explanations depends on the chronology of the increase in serotonin levels due to SERT blockage, the decrease due to 5-HT_1A_ autoreceptor activation, the potential SERT upregulation, and the relative magnitude of all these effects.

The negative relationship between citalopram-induced changes in ACC activation for fearful versus happy faces and self- versus other-attribution might also be seen from the perspective of passive coping. On the one hand, citalopram might have more influence on the ACC activation of people attributing emotional events primarily to themselves as there is more passive coping to develop. On the other hand, attributing such events to others or perceiving them as situational could already be seen as passive coping.

### The effects of depression and citalopram on neural fear responses

The clusters observed in the superior temporal lobe and cingulate sulcus show relative increases/decreases in activation after placebo/citalopram application, respectively, when contrasting fearful to happy faces. These changes originate from smaller (not significant, see supplement) and opposing changes in the contrasted emotions. Individuals with uninhibited temperament, constituting the majority, show increased dorsal ACC activation to expected fearful facial expressions [64]. Such expectations are also likely for the second run of our emotion identification task. Furthermore, the potential influence of citalopram on the processing of positive and threatening stimuli as discussed above might extend to other brain regions than ACC.

In the bilateral posterior insula, activation for fearful versus neutral stimuli relatively increased following placebo and decreased following citalopram application. A study using various modes of emotion evocation found increased bilateral posterior insula activation when comparing fearful to neutral stimuli [65]. The insula cortex might also play an important role in the development of MDD [66]. The relative reduction of the posterior insula activation we observed for fearful versus neutral stimuli under citalopram as compared to placebo might thus be a neural sign of the fast anxiolytic properties of citalopram observed in animal studies [67, 68].

Before challenge application, subjects with MDD compared to HCs showed higher activation to fearful versus scrambled faces in clusters reaching into the intraparietal and postcentral sulcus as well as along the superior frontal sulcus. A study of similar size found elevated activation to conditioned fear during the early extinction phase in patients with MDD, correlating with very high levels of repetitive negative thinking [69]. The effect covered parts of the postcentral and posterior parietal cortex. Meta-analytical results further indicate a general role of the inferior parietal cortex, extending to the intraparietal sulcus in human fear conditioning [44]. When viewing fearful versus calm faces, children and adolescents at risk for bipolar depression showed increased activation of the superior frontal cortex compared to HCs [70]. Together, these findings point towards dysfunctional processing of fear in patients with depression manifesting in increased neural responses of intraparietal, postcentral and superior frontal regions.

After at least three months of antidepressant treatment, patients with MDD showed an increase in activation around the calcarine/parieto-occipital fissure for fearful versus happy faces, which was primarily driven by a decrease for happy faces (see supplement). Previous findings include a decreased decoupling from the dorsomedial prefrontal cortex during positive versus neutral expectancy in MDD [71], a decreased resting activity in partially remitted depression compared to HCs [72], but also after compared to before vagus nerve stimulation [73]. Furthermore, an increased activation for happy versus irritating stimuli correlating with subjective positive effects of clomipramine in HCs was reported [74]. The reduction in calcarine/parieto-occipital fissure activation might thus be related to a treatment effect manifesting in the processing of positive rather than negative stimuli.

## Limitations

Some correlations being significant only for the difference between post- and pre-drug application scans and others only for the post-drug applications scans alone likely is a manifestation of the statistical bias-variance dilemma. While accounting for a daily bias, calculating the difference to the pre-drug applications increases the variance of the estimate, leading to variations in significance despite similar tests. Furthermore, the repetition of a task inevitably bears the risk of introducing habituation or sensitization effects. Despite the large sample size, several subjects had incomplete data, which we compensated by employing suitable models. In addition, the sample of patients with MDD was considerably smaller than that of HCs. Due to some necessary drug switches, the follow-up investigation is observational rather than showing pure escitalopram effects. To achieve the overall large sample size, data was collected over around 3.5 years. This duration implies that SERT values were affected by seasonal variation potentially introducing further variance [42]. While the reported correlations do not indicate large effect sizes, they are reasonable for brain-behavior and imaging genetics analyses and could be detected only based on our large sample size and targeted analyses [75, 76]. While we corrected our results for multiple comparisons, presenting different findings formally increases the chance that our work includes one or more false positives. Lastly, the opposing negative mediated influence of the number of L_A_ alleles on ACC activation via SERT BP_P_ and positive direct effect might indicate further mediating variables not considered here.

## Conclusion

We found that striatal SERT BP_P_ mediates the influence of the number of 5-HTTLPR/rs25531 L_A_ alleles on activation of the ACC when viewing fearful versus happy faces. A higher number of L_A_ alleles was associated with higher SERT BP_P_, which, in turn, was associated with lower ACC activation for fearful versus happy faces. Acute intravenous citalopram led to reduced activation in several brain regions involved in fear processing when viewing fearful faces, including the ACC. In the ACC, the citalopram-induced change in activation negatively correlated with self- but positively with other-attribution of emotional events. We interpret these findings as potential neural correlates of the proposed passive coping mechanism of action of SSRIs and the acute anxiolytic properties of citalopram. Moreover, lower fear-related ACC activation under acute citalopram as well as for higher SERT BP_P_ might indicate either a dependence on baseline SERT expression and citalopram-induced SERT upregulation, or less available serotonin in the synaptic cleft as common origin of our findings. Further studies are needed to disentangle the influence of SSRIs on the perception of different emotions and habituation. Lastly, the proposed passive coping mechanism of action of SSRIs warrants further investigation, which is required to cover multiple levels from molecular to psychological effects.

## Supplementary information

Supplement

## Data Availability

Data supporting the findings of this study are available from the corresponding author upon reasonable request.
