# Supplementary material for: A large-scale multimodal investigation of the interplay between the serotonergic system and emotion processing
Source: Transl Psychiatry. 2025 Jun 11;15:196. doi: 10.1038/s41398-025-03407-2 (PMC12159172; doi:10.1038/s41398-025-03407-2)
Supplement: Supplementary file 1 — Supplement [file 41398_2025_3407_MOESM1_ESM.docx]

Supplementary material for

A large-scale multimodal investigation of the interplay between the serotonergic system and emotion processing

Klöbl M^1,2^, Murgaš M^1,2^, Reed MB^1,2^, Silberbauer LR^1,2^, Hartmann AM^1,2^, Godbersen GM^1,2^, Gryglewski G^1,2,3^, Nics L^4^, Hahn A^1,2^, Rujescu D^1,2^, Hacker M ^4^, Lanzenberger R^1,2^

^1^ Department of Psychiatry and Psychotherapy, Medical University of Vienna, Vienna, Austria

^2^ Comprehensive Center for Clinical Neurosciences and Mental Health, Medical University of Vienna, Vienna, Austria

^3^ Child Study Center, Yale University, New Haven, CT, USA

^4^Department of Biomedical Imaging and Image-guided Therapy, Division of Nuclear Medicine, Medical University of Vienna, Vienna, Austria

# Supplementary Materials and Methods

## Study design

MRI scans with crossover, randomized, placebo-controlled infusion of the study medication were separated by one week to allow for sufficient washout. The study medication consisted of 8 mg citalopram dissolved in 8 ml saline or 16 ml saline alone over the course of 8 minutes.

A pilot study was conducted in order to optimize the experimental design and allow for adaptations of our instruments (e.g., the emotion identification task) to improve sensitivity to our research questions. Since we did not want to expose patients with MDD to a suboptimal research design, only healthy subjects were enrolled in the pilot study. In the case of the emotion identification task, we deemed the pilot and the optimized version sufficiently similar to merge their data for the analyses presented in this work while still adjusting our statistical analyses for the study phase.

## Subjects

Subjects were excluded in case of positive drug urine or pregnancy tests at the screening visit or on the scanning days. Patients with MDD were not included in case of prior psychopharmacological treatments within the last three months excluding short-term benzodiazepine or antihistamine intake. For additional information on inclusion and exclusion criteria see ^1-4^.

## Functional magnetic resonance imaging acquisition and analysis

Recordings of the emotion identification task comprised 235 frames in the main and 230 frames in the pilot phase of the study. Preprocessing of the task recordings was performed using Statistical Parametric Mapping, version 12 (SPM12; https://www.fil.ion.ucl.ac.uk/spm/software/spm12/) with standard parameters unless explicitly stated: Images were slice-timing corrected to the temporally middle slice, all task recordings of the same subject were realigned together with quality set to 1, and normalization was performed to the standard space defined by the Montreal Neurological Institute. Interpolation was set to the highest order. We applied wavelet despiking ^5^ to remove non-linear artifacts with a “threshold” of 25 due to higher noise levels of unsmoothed and GRAPPA-accelerated data. Chain search was set to “harsh” for increased sensitivity to slow artifacts. Finally, images were smoothed with implicit masking to retain the edges of the brain. The four task regressors were convolved with the hemodynamic response function. Nuisance regressors comprised the Friston-24 model ^6^ and an adaptive CompCor approach ^7,8^.

## Whole-brain fMRI analysis

Group comparisons of the pre-drug application runs, run-by-condition, and group-by-run-by-condition interaction effects were estimated on overdetermined models (i.e., the way SPM12 creates design matrices). To properly set contrast on the follow-up scans comparing them to the pre-drug application scans of the HC and MDD group as well as the post-drug application run in the verum condition of the MDD group, additional underdetermined models were created without adjusting for order and habituation as these were necessarily collinear to the follow-up regressor.

To calculate the parameters for multiplicity correction, AFNI’s 3dFWHMx with detrending was used on the smoothed fMRI time series. The median parameters of the autocorrelation function were then fed into 3dClustSim. Bisided results for clusters defined by touching voxel faces (NN1) are reported with a primary threshold of p < 0.001 uncorrected and p < 0.05 corrected, both two-sided ^9^. Multiplicity was corrected for using Sidak adjustment. Subsequent LME models included the same terms as the whole-brain models with additional random intercepts, conditions, and runs per subject.

## [^11^C]DASB positron emission tomography analysis

BP_P_ (placebo and verum values separately) and occupancy were harmonized using the ComBat algorithm adjusting for subject group (no patients with MDD in the pilot phase) and order of drug application, which showed a relevant difference between pilot and main study ^10^.

In addition to thalamus and striatum, we extracted the signal from the dorsal and median raphe nuclei as origins of serotonergic signaling.

## 5-HTTLPR/rs25531 genotyping

DNA was isolated from 9 ml Ethylene-Diamine-Tetraacetic-Acid (EDTA) whole-blood samples using the QIAamp DNA Blood Maxi Kit (Qiagen, Hilden, Germany). PCR products were divided and also used for determination of the L_A_ and L_G_ variants. Here, 15 µl were digested with 2 U MspI resulting in different fragment lengths for the L_A_ and L_G_ variants which were also separated by agarose gel electrophoresis.

## Psychometric assessment and evaluation

The combination of HAM-D, MARDS, and BDI allowed us to derive complementary aspects of symptom severity (observed mood, cognitive symptoms, neurovegetative symptoms ^11^). We used these for descriptive analyses (see Table S1) and correlations with whole-brain follow-up effects.

For the IPSAQ-R, subjects have to imagine 32 predefined positive and negative situations involving them and a friend and write down potential causes for the events. Then subjects have to state for each cause to what percentage it depends on them, other people, or the situation.

Missing values were imputed as previously described ^12,13^. In short, the single items of the scores were normalized to the range (-1, 1), and Fisher-z-transformed to avoid negative values after imputation. Whenever possible, we linearly interpolated missing depression scores in the MDD group from temporally neighboring values. We employed data augmentation for the remaining missing depression scores of the MDD group (more data points than variables, 1.66% missing) and trimmed score regression (more variables than data points) in all other cases (3.16% missing for HC depression scores, 32.71% for MDD, and 5.83% for HC IPSAQ-R). Imputation was performed over all depression scores and items simultaneously but separately for subject groups and the IPSAQ-R. We chose the number of principal components according to the minimum predicted residual error sum of squares (PRESS). Reversing the transformation steps yielded the imputed data on its original scale.

## Multimodal analysis

Correlation coefficients of LME regressors were derived from the “drop in R-squared” measure.

Based on previous research from our group indicating the relevance of adjusting for raphe SERT levels in MDD ^14^, we reran our fMRI activation/SERT binding models with a covariate for the raphe nuclei. Furthermore, we analyzed whether the thalamus/striatum signal only mediates the influence of the raphe nuclei on fMRI activation.

Due to strong correlations of SERT binding between the striatum and the thalamus as well as between the dorsal and median raphe nuclei, respectively (r = [0.67, 0.97]), and in order to reduce the number of comparisons, the first principal components of the PET data were used for the above analyses, i.e., one principal component for striatum/thalamus and one for raphe nuclei. For the same reasons (mean |r| = 0.46), we used the first two principal components of the IPSAQ-R scales.

|  | **HC main** | **MDD main** |
| --- | --- | --- |
| HAM-D | baseline: 0 ± 0 | baseline: 19.67 ± 3.33  follow-up: 5.00 ± 4.50 |
| MADRS | baseline: 0 ± 0 | baseline: 27.50 ± 6.58  follow-up: 8.00 ±13.00 |
| BDI | baseline: 0 ± 0 | baseline: 25.33 ± 10.63  follow-up: 9.50 ± 15.00 |
| Observed mood |  | baseline: 26.03 ± 5.25  follow-up: 7.63 ± 9.46 |
| Cognitive symptoms |  | baseline: 23.88 ± 8.79  follow-up: 8.09 ± 11.57 |
| Neurovegetative symptoms |  | baseline: 11.01 ± 7.39  follow-up: 3.34 ± 6.23 |

Table S1: Overview of depressive symptom severity using questionnaires and derived measures. HAM-D: Hamilton depression rating scale, MADRS: Montgomery-Åsberg depression rating scales, BDI: Beck’s depression inventory. Observed mood, cognitive, and neurovegetative symptoms were derived according to ^11^

### Mediation of striatal/thalamic BP_P_ influence on anterior cingulate/medial prefrontal cortex activation

The fMRI activation cluster that was further investigated as described below stretched from the ACC into the mPFC. For the sake of readability, it is only referred to as “ACC”. For testing a potentially striatum/thalamus-mediated effect of the raphe BP_P_ influence on ACC activation after challenge application for fearful versus happy faces, we used the following setup in the R package “mediation” ^15^: Outcome and mediator model were defined as linear mixed effects models. The outcome model regressed the ACC activation on the first principal component (denoted as “PC1” in the formulas below) of striatum/thalamus BP_P_ and was adjusted for the study design variables phase (pilot, main), group (HC, MDD), substance (placebo, citalopram) and unwanted influences of habituation, order of drug application, subject sex and age. The mediator model regressed the first principal component of striatum/thalamus BP_P_ on the first principal component of dorsal/median raphe BP_P_. Both models contained a random intercept per subject to account for the inclusion of placebo and citalopram values.

Outcome model:

$$ACC activation \sim phase+group+substance+PC1\left( {{striatum}/{thalamus}BP}_{P} \right)+PC1\left( {{dorsal}/{medial raphe}BP}_{P} \right)+habituation+order+age+sex+\left( 1 | subject \right)$$

Mediator model:

$$PC1\left( {{striatum}/{thalamus}BP}_{P} \right) \sim PC1\left( {{dorsal}/{medial raphe}BP}_{P} \right)+\left( 1 | subject \right)$$

The mediation analysis was run on 213 data points with 10000 simulations and quasi-Bayesian approximated, bias-corrected and accelerated confidence intervals.

### Mediation of 5-HTTLPR/rs25531 influence on anterior cingulate/medial prefrontal cortex activation

We conducted testing of striatal BP_P_ mediation of the 5-HTTLPR/rs25531 polymorphism and ACC activation relationship using simpler models since only the placebo condition was relevant. Based on previous literature ^16,17^ and the correlations between 5-HTTLPR/rs25531 L_A_ allele frequency and striatum BP_P_ under placebo as well as median/dorsal raphe BP_P_ and ACC activation for fearful versus happy faces after either placebo or citalopram application, we focused on striatum BP_P_ and the post-placebo activation. Also based on literature, we conditioned the test on subject age ^18^. With variable names as above and the L_A_ allele frequency denoted by #L_A_, we set up the following models:

Outcome model:

$$ACC activation \sim group+{striatum BP}_{P}+{\#L}_{A}+order+age+sex$$

Mediator model:

$${striatum BP}_{P} \sim\#L_{A}$$

The mediation analysis was run on 94 data points with 10000 nonparametric bootstrap draws and bias-corrected and accelerated confidence intervals. For comparison only, we rerun the model with the average of both pre-drug application and the post-placebo application run (i.e., all runs without citalopram influence).

# Supplementary Results

## Symptom improvements and drug dosage

HAMD, MADRS, and BDI scores at follow-up were collected for 28 of the 29 subjects with follow-up fMRI scans. The remission numbers for the respective scores was as follows: HAMD ≤ 7: 22 (78.57%), MADRS ≤ 10: 19 (67.86%), BDI ≤ 9: 15 (53.57%). A response defined as at least 50% symptom reduction (calculated as average over the pre-screening and both PET/fMRI assessments) was achieved for the following number of patients: HAMD: 23 (82.14%), MADRS: 22 (82.14%), BDI: 17 (60.71%). The number of responders include the remitters.

Due to insufficient response, four subjects were switched to mirtazapine, two to venlafaxine, one again from mirtazapine to duloxetine, and one again from mirtazapine to venlafaxine during the follow up period. The subject switched from mirtazapine to venlafaxine had discontinued venlafaxine at follow-up due to adverse interactions. At follow-up, two subjects received 30 mg mirtazapine, two received 150 mg mirtazapine, and two received 60 mg duloxetine. The rest of the subjects received 10 to 20 mg escitalopram with a median dose of 12.5 mg.

## Emotion identification task

The baseline activation and deactivation patters for all task contrasts are shown in Figure S1 (both pre-drug application scans adjusted for age, sex, study phase, habituation and potential carryover effects). As expected, all stimuli significantly activated visual regions. The facial but not the scrambled stimuli also significantly activated subcortical regions including the amygdalae and parts of the thalamus as well as the anterior insula.

The post-drug application runs started 53.45 ± 3.53 minutes after infusion and both runs were separated by 75.12 ± 3.72 minutes (median ± interquartile range). We performed top-down testing of the contrasts, starting with the difference of fearful and happy faces. Based on our initial findings, we subsequently tested fearful and happy faces separately versus neutral faces, fearful versus scrambled face and fearful faces alone. Additional multiplicity correction was thus conducted for five contrasts.

Figure S2 shows the separate base contrasts of the significant effects identified when subtracting the contrasts from each other (e.g., the separate fearful and happy contrasts for the fearful versus happy results).

| Negative | 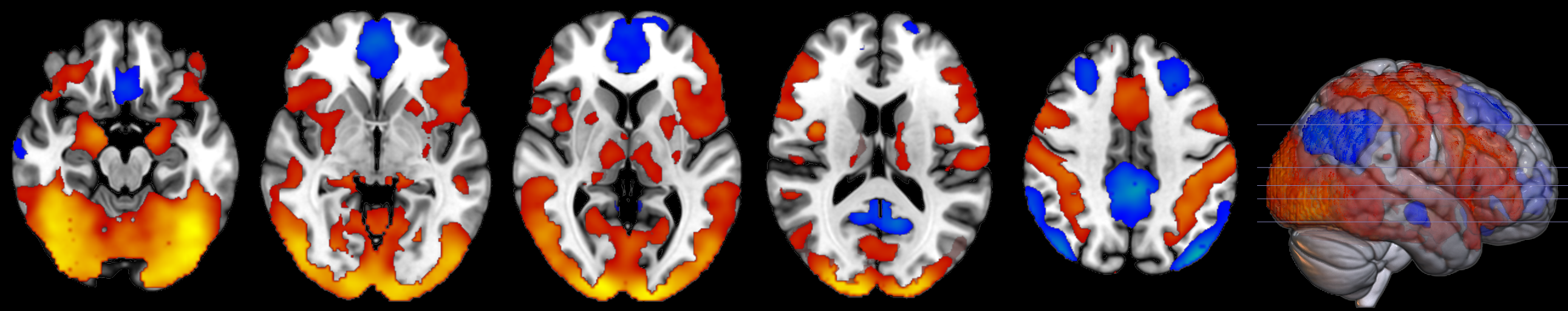 |
| --- | --- |
| Positive | 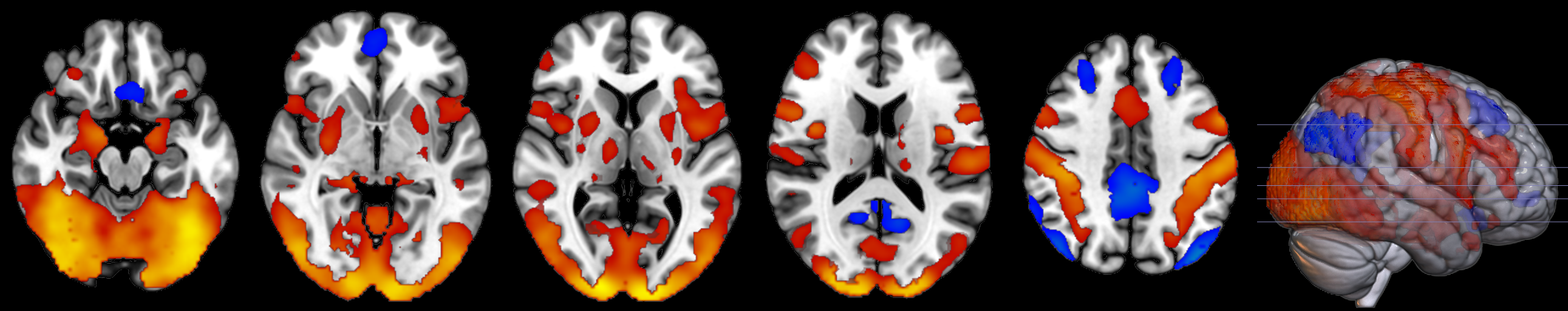 |
| Neutral | 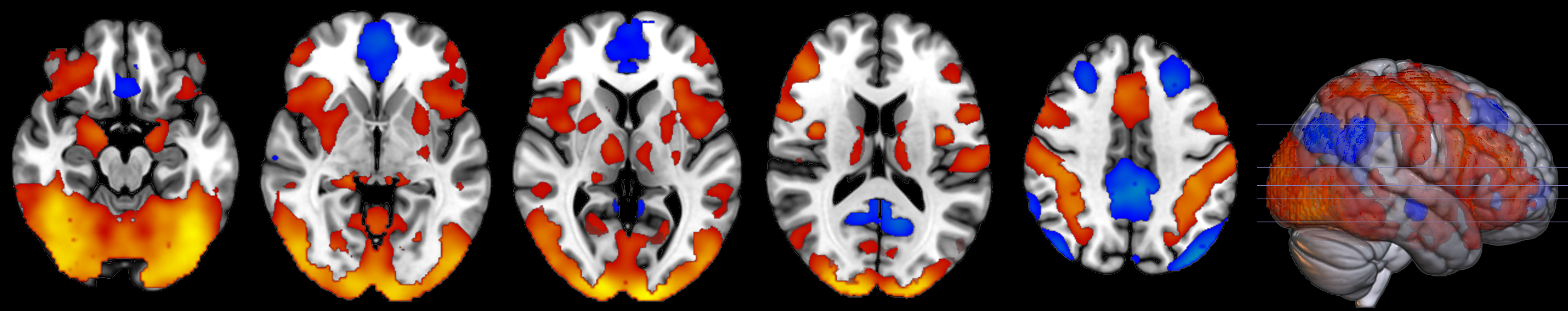 |
| Scrambled | 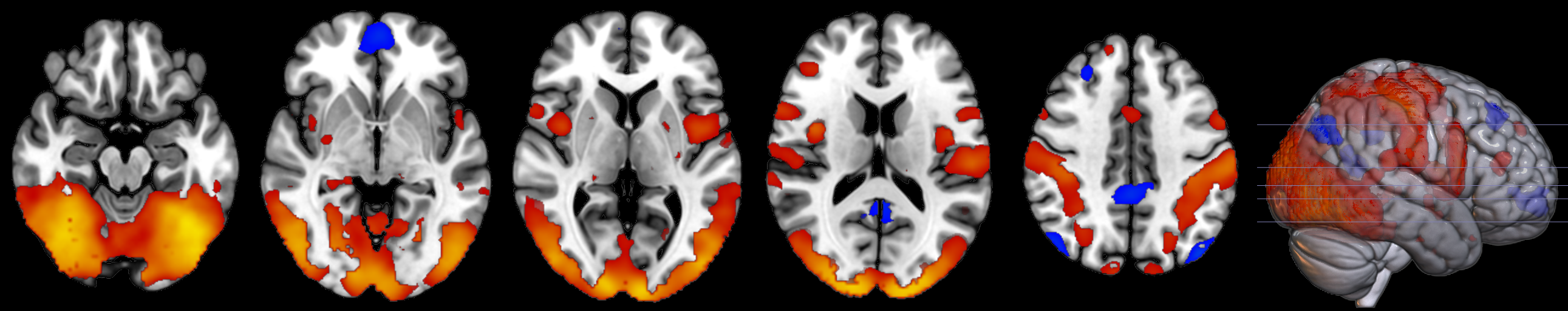 |
| Negative – positive | 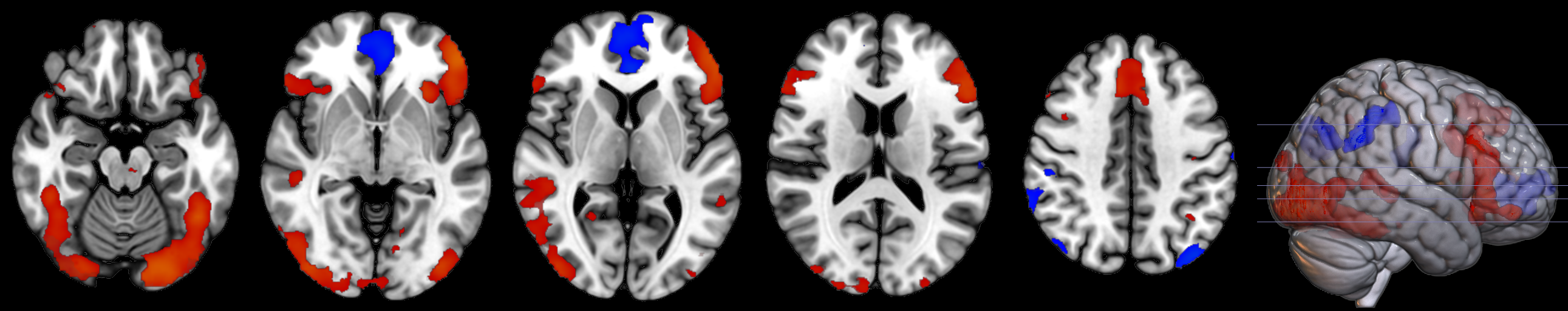 |
| Negative – neutral | 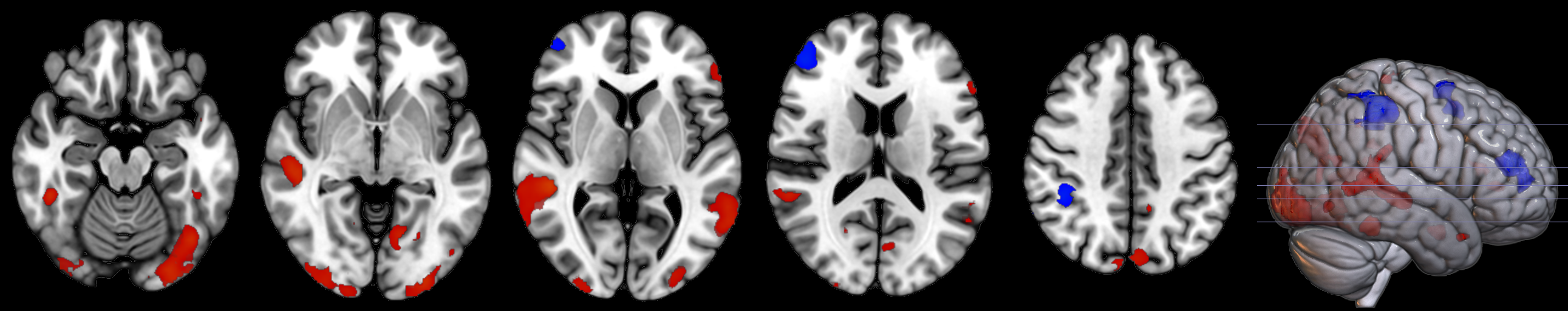 |
| Positive –  neutral | 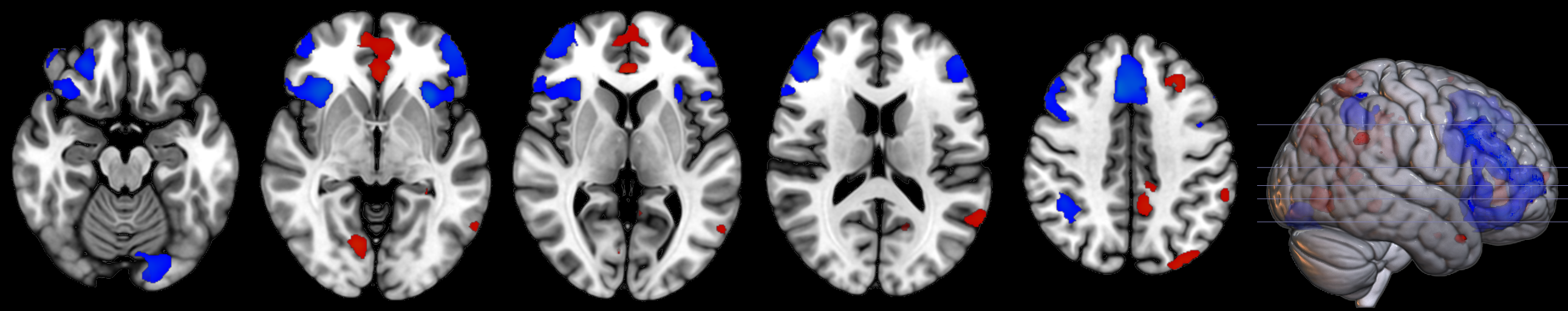 |
| Negative – scrambled | 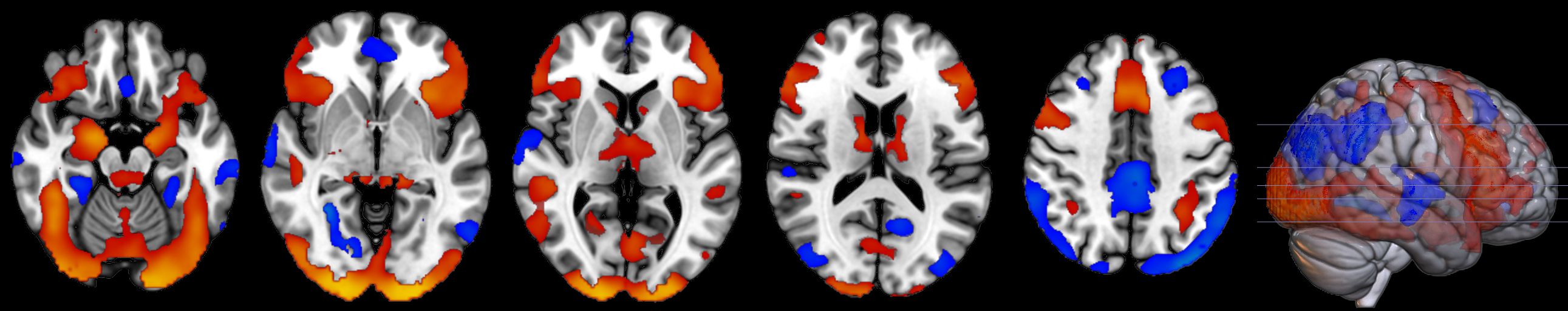 |
| Positive – scrambled | 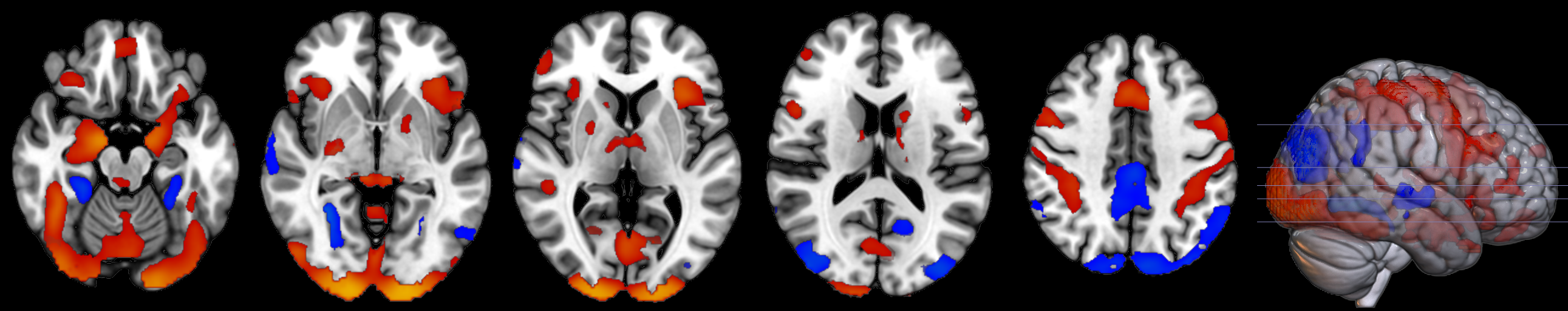 |
| Neutral – scrambled | 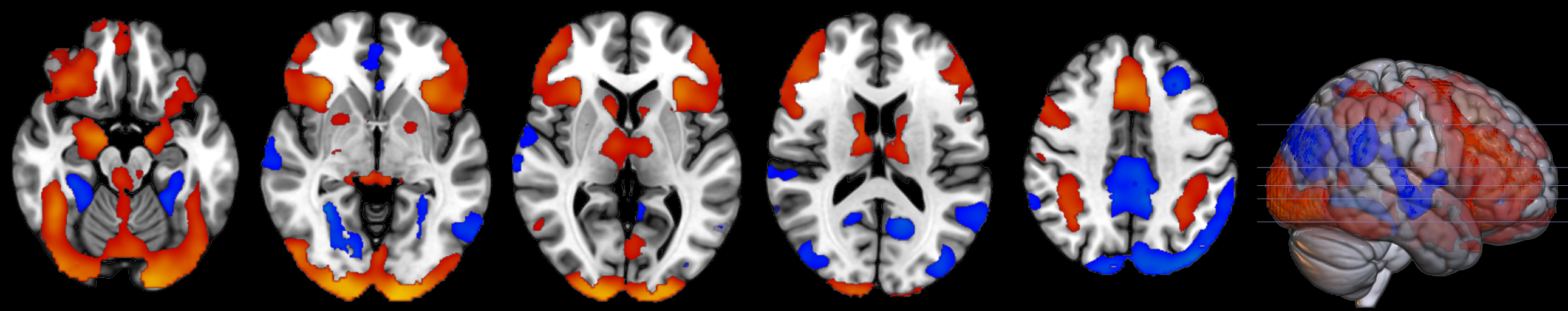 |
| z-values | 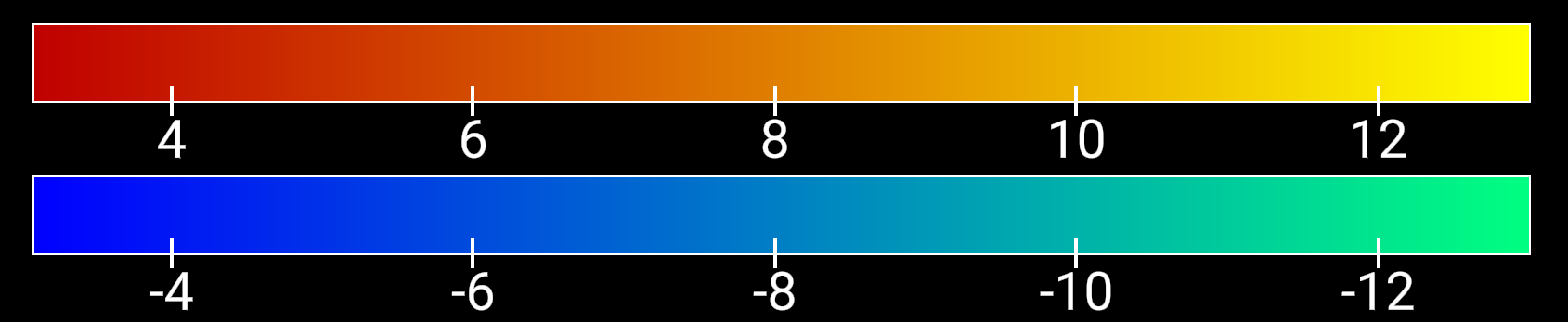 |

Figure S1: Baseline activation (warm colors) and deactivations (cold colors) of each contrast. z-maps are display at cluster-level with a primary threshold of p < 0.001 and a familywise error correction of p < 0.05, two-sided. Axial slices shown at z = -18, -4, 4, 15, 41.

## Relation between brain activation and serotonin transporter binding

The first principal component of the forebrain regions (striatum, thalamus) for [^11^C]DASB explained 98.42% and 97.66% variance for binding potential and occupancy, respectively. The explained variance was 93.11% and 83.70% for the dorsal and mesial raphe nuclei. Similar effect sizes within the single drug conditions indicated that the negative correlation between BP_P_ and ACC activation for fearful versus happy faces is not driven by a residual difference (both r = -0.18).

The negative correlation between striatum/thalamus SERT BP_P_ and ACC activation for fearful versus happy faces across both drug conditions for the post-application runs became non-significant when including the raphe signal in the model. In light of these results, further analyses to test whether striatum/thalamus mediates the influence of the raphe BP_P_ on ACC activation were performed. No significant separable direct and mediation effects but a significant total effect of BP_P_ on ACC activation for fearful versus happy faces was found (p = 0.0012).

## Relation between brain activation and attribution styles

We tested the first two principal components of the IPSAQ-R for a relationship with ACC activation as they explained similar amounts of variance (44.88% and 38.54%, respectively). We found no significant correlation for the second component mainly described situational attribution in contrast to the alternatives (except for positive self-attribution). Of note, due to the potential answers summing up to 100%, the components were partly redundant. However, elimination of the situational attribution percentages, and thus redundancy, yielded comparable results (first principal component explaining 67.11% of the total variance again comprising the difference between self- and other-attribution (r = -0.27, p_SiSi_ = 0.0168)).

| 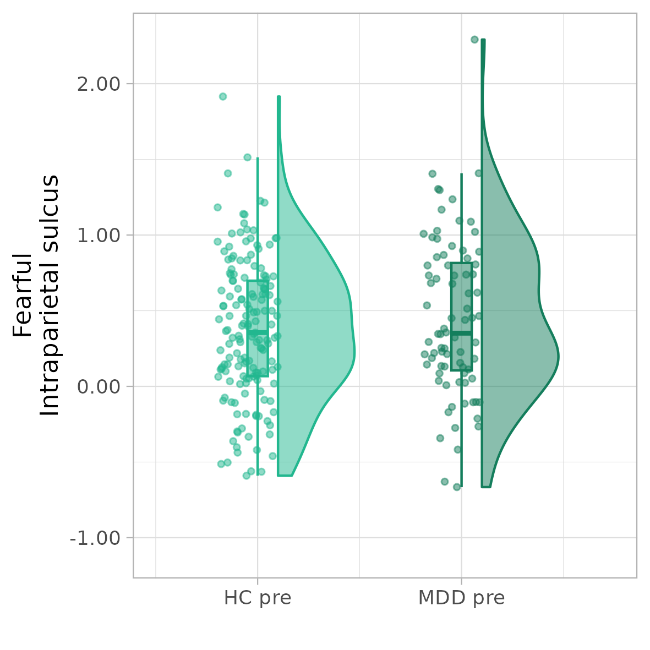 | 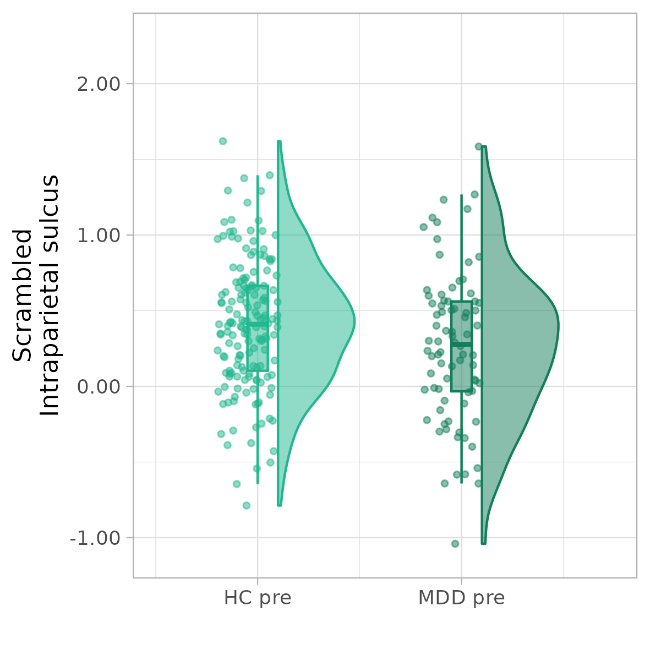 |
| --- | --- |
| 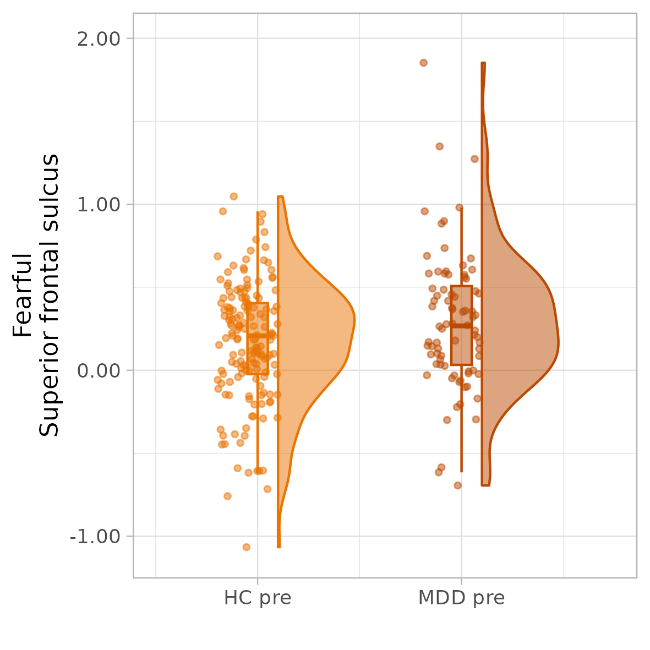 | 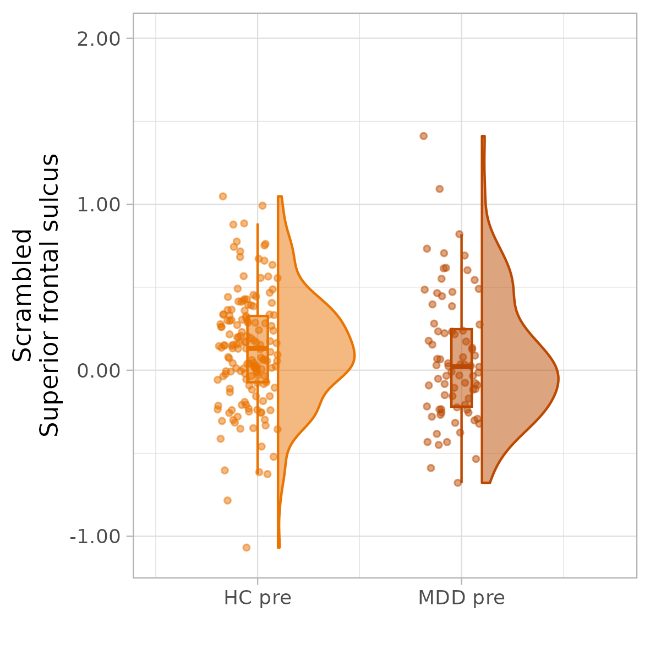 |
| 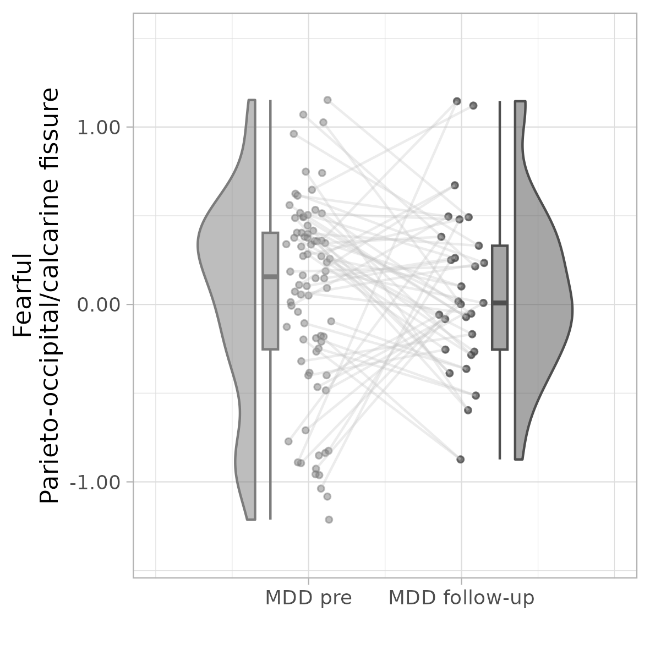 | 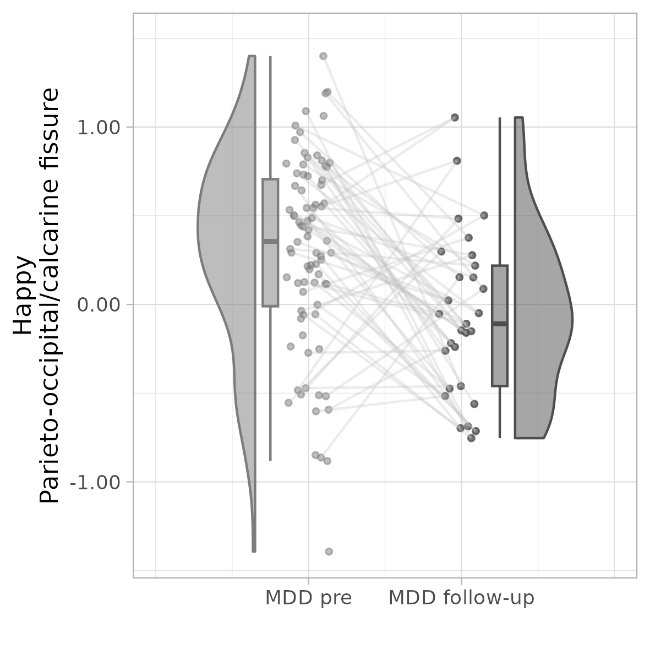 |
| 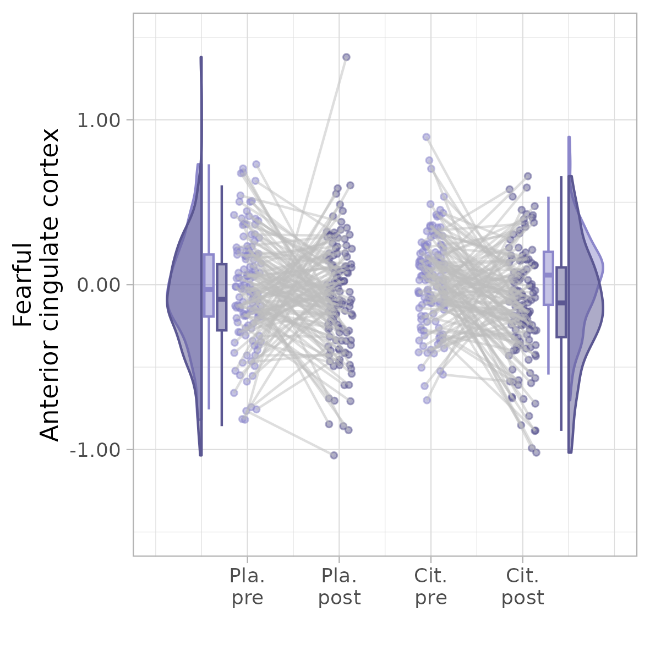 | 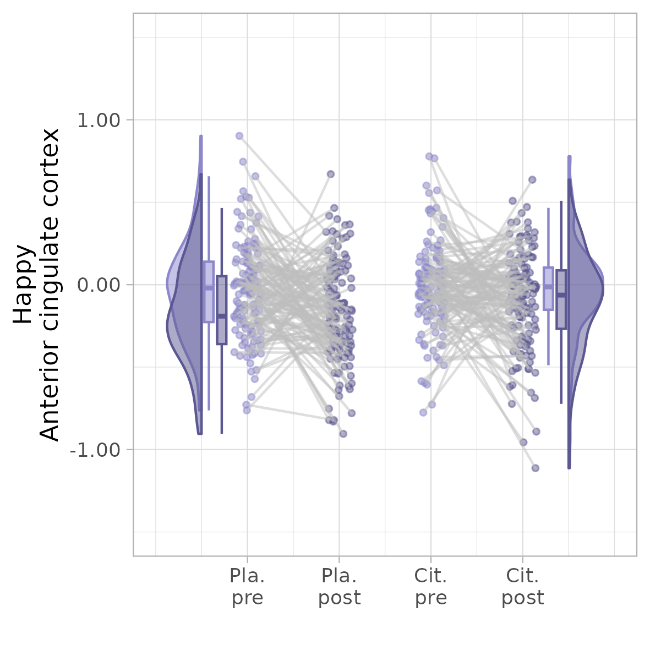 |
| 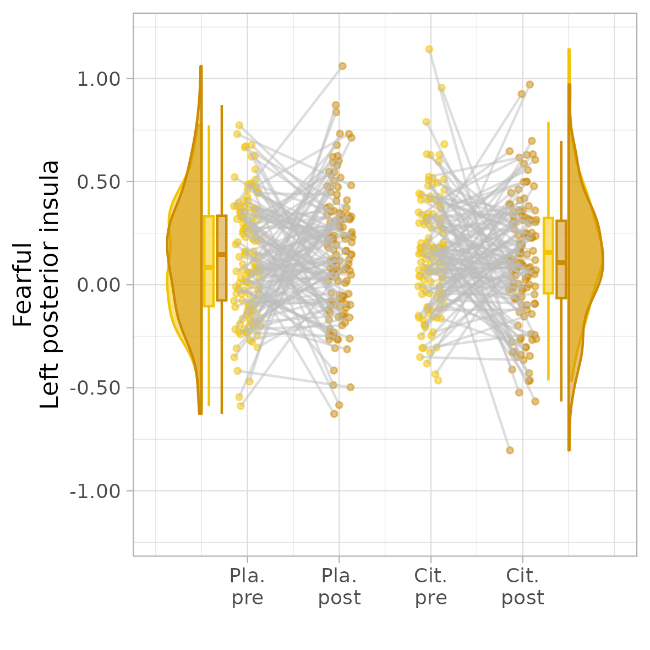 | 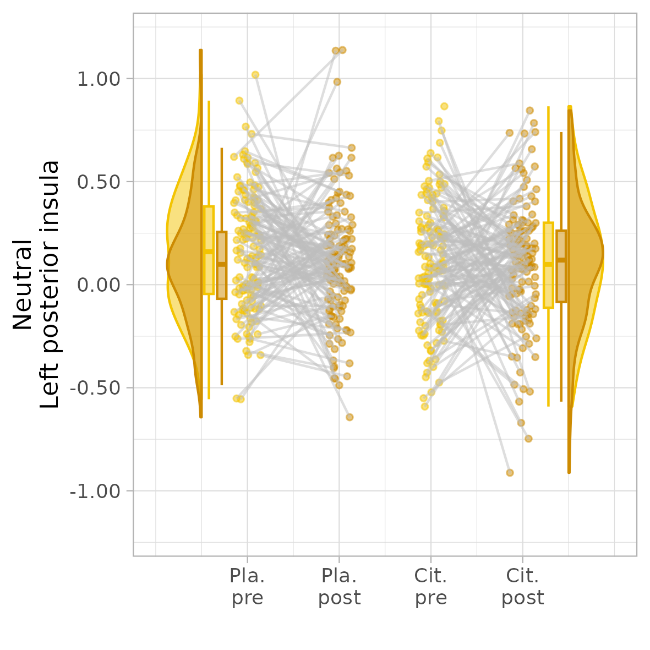 |
| 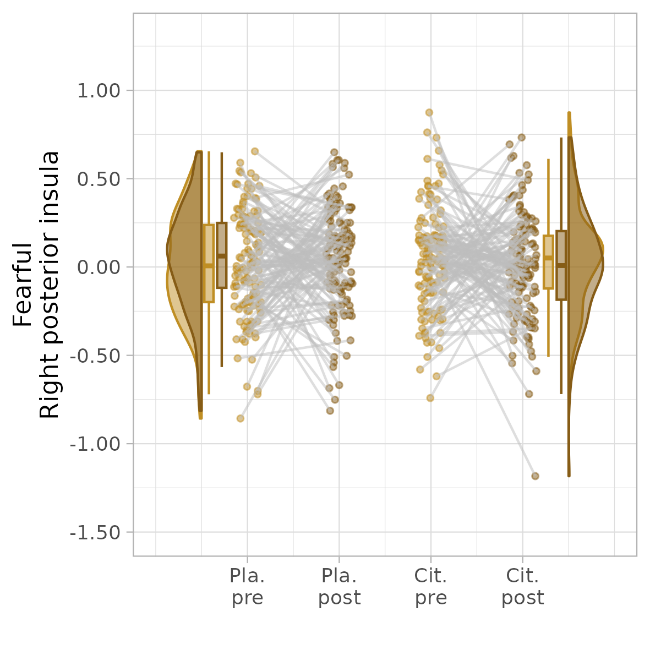 | 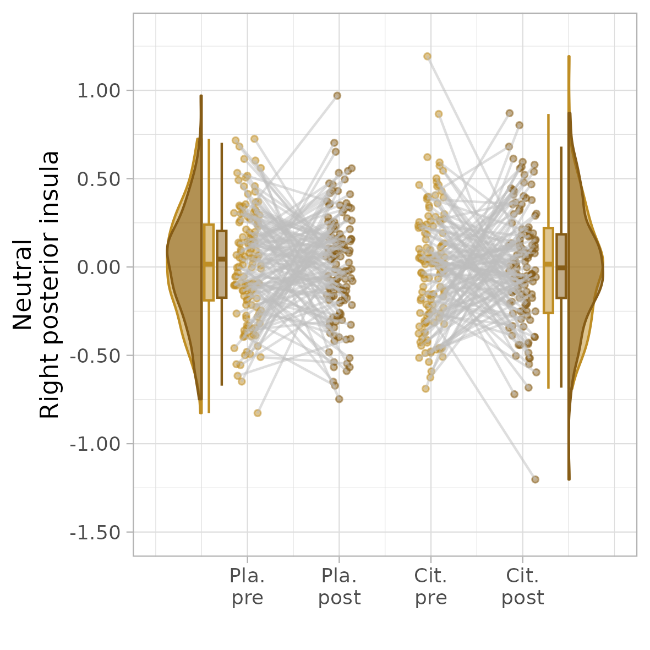 |
| 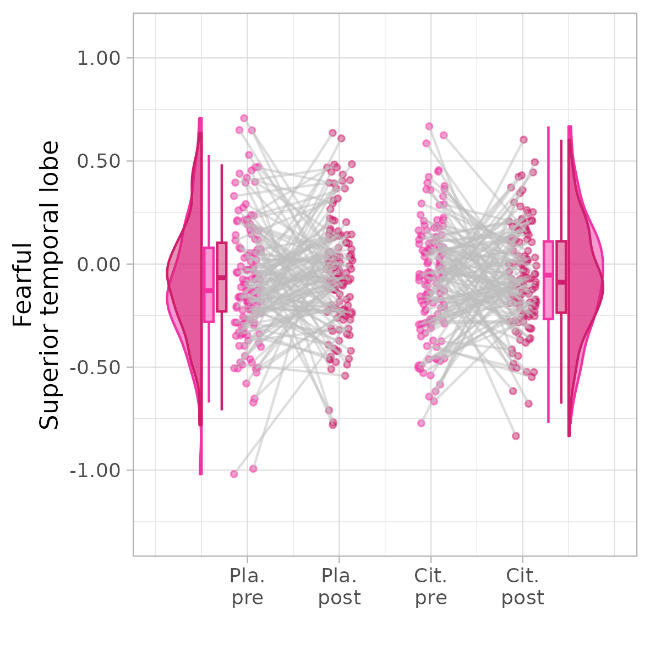 | 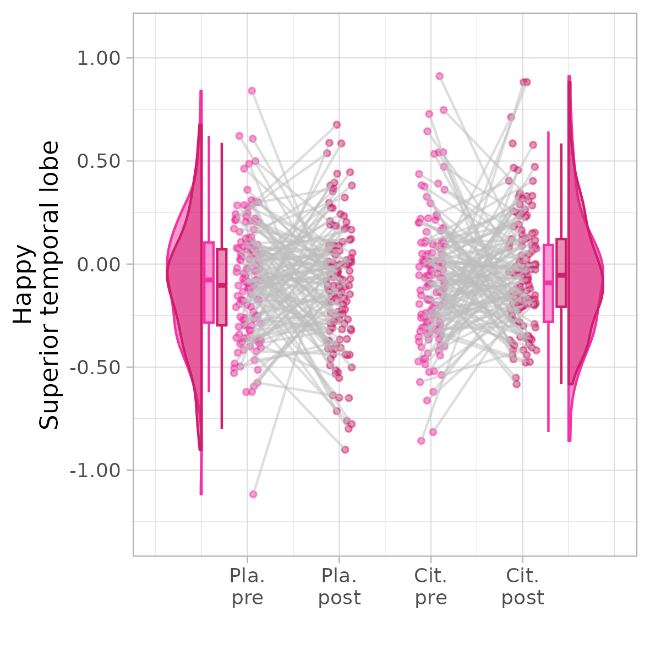 |
| 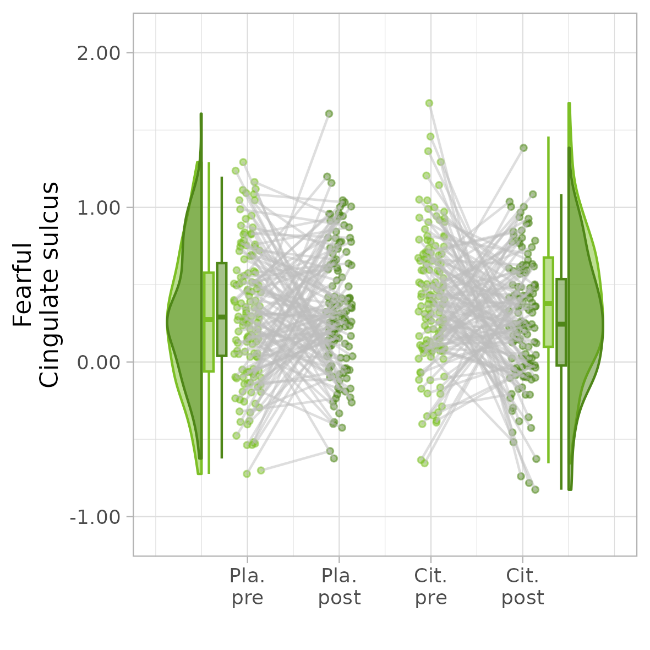 | 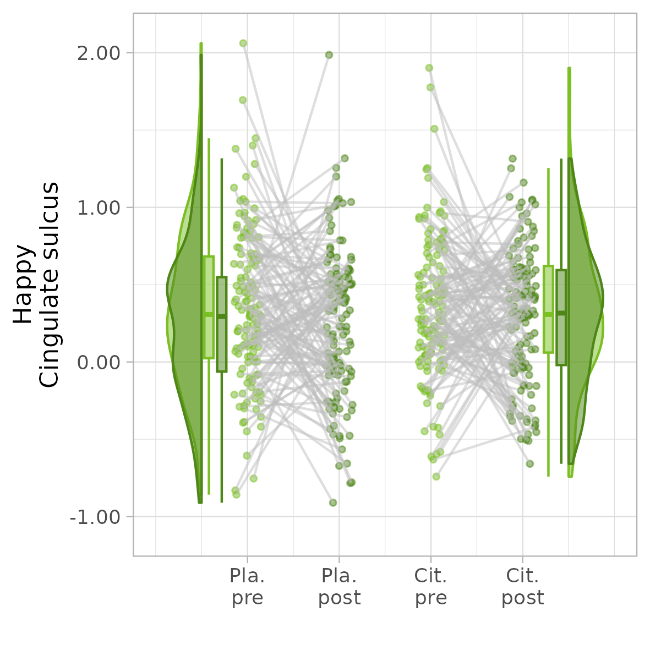 |

Figure S2: Comparisons of base contrasts leading to significant effects when subtracted.

| **Name** | **Estimate** | **SE** | **t-value** | **DF** | **p_uncorr_-value** | **p_SiSi_-value** |
| --- | --- | --- | --- | --- | --- | --- |
| intercept | 0.21919 | 0.04946 | 4.4317 | 205 | 1.52E-05 | 0.00020879 |
| pilot | 0.065101 | 0.057085 | 1.1404 | 205 | 0.25544 | 0.98252 |
| MDD | -0.078753 | 0.037938 | -2.0758 | 205 | 0.039159 | 0.42191 |
| citalopram | -0.39271 | 0.091631 | -4.2857 | 205 | 2.80E-05 | 0.0003842 |
| PC1(striatum/thalamus BP_P_) | -0.10153 | 0.03283 | -3.0927 | 205 | 0.0022593 | 0.030554 |
| habituation | -0.0216 | 0.03473 | -0.62193 | 205 | 0.53468 | 0.99997 |
| order | -0.069101 | 0.036784 | -1.8786 | 205 | 0.061725 | 0.58275 |
| age | 0.00097546 | 0.0020684 | 0.47159 | 205 | 0.63772 | >0.9999 |
| sex | 0.011184 | 0.038168 | 0.29302 | 205 | 0.7698 | >0.9999 |

Table S2: Correlation analysis of the first principal component (PC1) of striatum/thalamus BP_P_ with the activation in the anterior cingulate/medial prefrontal cortex for fearful versus happy face after either placebo or citalopram application. The analysis was adjusted for participation in the pilot study, a diagnosis of major depressive disorder (MDD), receiving citalopram, habituation and order effects, age, and sex. Raw p-values (p_uncorr_) were corrected for multiple testing using sine-similarity (p_SiSi_).

## Relation between brain activation and serotonin transporter expression

Table S2 shows the details of the correlation analysis of ACC activation for fearful versus happy faces after receiving either placebo or citalopram against striatum/thalamus BP_P_. To account for the dependence of placebo and citalopram cans of the same subjects, we used a linear mixed effects model.

Table S3 shows the detailed results of the mediation analysis of raphe influences on the relationship between striatum/thalamus BP_P_ and ACC activation when viewing fearful versus happy faces. There was a significant total effect (p = 0.0012) of raphe and striatal/thalamic BP_P_ on the ACC activation but no separable mediation and direct effects.

Testing for mediation effects of striatum BP_P_ on the relationship between 5-HTTLPR/rs25531 polymorphism and ACC activation after placebo application (Table S4) revealed a significant mediation effect (p = 0.0430) and a direct effect falling short of significance (p = 0.0950). Averaging ACC activation over all runs without citalopram influence (Table S5), reverses the pattern of significance while also revealing a significant total effect (mediation: p = 0.0830, direct: p = 0.0060, total: p = 0.0230). For interpretability, the fMRI activation and SERT BP_P_ values for these models were standardized prior to mediation analysis.

|  | **Estimate** | **95% CI Lower** | **95% CI Upper** | **p-value** |
| --- | --- | --- | --- | --- |
| Total | -0.1114 | -0.1781 | -0.04 | 0.0012 |
| ACME | -0.0674 | -0.1561 | 0.02 | 0.1316 |
| ADE | -0.0439 | -0.1244 | 0.04 | 0.2910 |
| Prop. Mediated | 0.6038 | -0.2825 | 1.47 | 0.1316 |

Table S3: Detailed results of the mediation analysis of raphe binding potential on the relationship between striatum/thalamus binding potential on the activation of the anterior cingular/medial prefrontal cortex when viewing fearful versus happy faces after placebo or citalopram application.

|  | **Estimate** | **95% CI Lower** | **95% CI Upper** | **p-value** |
| --- | --- | --- | --- | --- |
| ACME | -0.0884 | -0.2472 | -0.01 | 0.0430 |
| ADE | 0.2676 | -0.0479 | 0.59 | 0.0950 |
| Total Effect | 0.1791 | -0.1220 | 0.50 | 0.2550 |
| Prop. Mediated | -0.4936 | -65.5534 | -0.05 | 0.2850 |

Table S4: Mediation analysis of the 5-HTTLPR/rs25531polymorphism L_A_ allele frequency on the relationship between striatum binding potential on the activation of the anterior cingular/medial prefrontal cortex when viewing fearful versus happy faces after placebo application.

|  | **Estimate** | **95% CI Lower** | **95% CI Upper** | **p-value** |
| --- | --- | --- | --- | --- |
| ACME | -0.0755 | -0.2171 | 0.00 | 0.0830 |
| ADE | 0.3956 | 0.1086 | 0.69 | 0.0060 |
| Total Effect | 0.3201 | 0.0460 | 0.61 | 0.0230 |
| Prop. Mediated | -0.2358 | 0.1475 | 1940.06 | 0.1050 |

Table S5: Mediation analysis of the 5-HTTLPR/rs25531polymorphism L_A_ allele frequency on the relationship between striatum binding potential on the activation of the anterior cingular/medial prefrontal cortex when viewing fearful versus happy faces averaged over the pre-drug and post-placebo application runs.

## Relation between brain activation and attribution styles

| **Name** | **Estimate** | **SE** | **t-value** | **p_uncorr_-value** | **p_SiSi_-value** |
| --- | --- | --- | --- | --- | --- |
| intercept | -0.20036 | 0.062434 | -3.2091 | 0.0018107 | 0.0057887 |
| MDD | -0.15319 | 0.10699 | -1.4318 | 0.15544 | 0.41793 |
| PC1(IPSAQ-R) | -0.090177 | 0.031595 | -2.8542 | 0.0052861 | 0.016835 |
| order | -0.13889 | 0.10883 | -1.2762 | 0.20496 | 0.52037 |
| age | -0.0066356 | 0.0061408 | -1.0806 | 0.28259 | 0.65489 |
| sex | -0.020114 | 0.10882 | -0.18483 | 0.85375 | 0.99788 |

Table S6: Correlation analysis of the first principal component (PC1) of the Internal, Personal and Situational Attributions Questionnaire – revised (IPSAQ-R) with the activation in the anterior cingulate/medial prefrontal cortex for fearful versus happy faces under citalopram versus placebo corrected for the pre-drug application runs. The analysis was adjusted for a diagnosis of major depressive disorder (MDD), order of drug conditions, age, and sex. Raw p-values (p_uncorr_) were corrected for multiple testing using sine-similarity (p_SiSi_).

# Supplementary Discussion

The observed effect in the ACC for fearful versus happy faces consist of strong reductions in the activation to happy faces after receiving placebo and to fearful faces after receiving citalopram. While a reduced fear-related activation in ACC under (es-)citalopram is known ^19,20^, the decrease for happy faces only under placebo might result from habituation together with a positive bias induced by SSRIs ^21,22^. An early study on emotional habituation further suggested a sustained supply of resources for processing stimuli signaling threat but a diminished supply for safety ^23^. If citalopram prevents the decrease of neural resources allocated to the analysis of known safety, this might indeed manifest as increased (passive) focus on positivity.

There seem to be a direct effect of the 5-HTTLPR/rs25531 polymorphism on ACC activation in response to fearful versus happy faces as well as a mediation by SERT BP_P_. Given the slightly varying results, depending on which runs of the emotion identification task were included in the analysis, there might either be small effect sizes or the influences are difficult to separate. In both cases, large sample sizes will be necessary to wholly disentangle the influences of the serotonergic system on emotion processing.

# Supplementary References

1 Klöbl, M. *et al.* Predicting Antidepressant Citalopram Treatment Response via Changes in Brain Functional Connectivity After Acute Intravenous Challenge. *Front Comput Neurosci* **14**, 554186, doi:10.3389/fncom.2020.554186 (2020).

2 Gryglewski, G. *et al.* Modeling the acute pharmacological response to selective serotonin reuptake inhibitors in human brain using simultaneous PET/MR imaging. *Eur Neuropsychopharmacol* **29**, 711-719, doi:10.1016/j.euroneuro.2019.04.001 (2019).

3 Seiger, R. *et al.* The Influence of Acute SSRI Administration on White Matter Microstructure in Patients Suffering From Major Depressive Disorder and Healthy Controls. *Int J Neuropsychopharmacol* **24**, 542-550, doi:10.1093/ijnp/pyab008 (2021).

4 Silberbauer, L. R. *et al.* ABCB1 variants and sex affect serotonin transporter occupancy in the brain. *Mol Psychiatry* **27**, 4502-4509, doi:10.1038/s41380-022-01733-1 (2022).

5 Patel, A. X. *et al.* A wavelet method for modeling and despiking motion artifacts from resting-state fMRI time series. *Neuroimage* **95**, 287-304, doi:10.1016/j.neuroimage.2014.03.012 (2014).

6 Friston, K. J., Williams, S., Howard, R., Frackowiak, R. S. & Turner, R. Movement-related effects in fMRI time-series. *Magn Reson Med* **35**, 346-355, doi:10.1002/mrm.1910350312 (1996).

7 Behzadi, Y., Restom, K., Liau, J. & Liu, T. T. A component based noise correction method (CompCor) for BOLD and perfusion based fMRI. *Neuroimage* **37**, 90-101, doi:10.1016/j.neuroimage.2007.04.042 (2007).

8 Klöbl, M. *et al.* Reinforcement and Punishment Shape the Learning Dynamics in fMRI Neurofeedback. *Front Hum Neurosci* **14**, 304, doi:10.3389/fnhum.2020.00304 (2020).

9 Chen, G. *et al.* A tail of two sides: Artificially doubled false positive rates in neuroimaging due to the sidedness choice with t-tests. *Hum Brain Mapp* **40**, 1037-1043, doi:10.1002/hbm.24399 (2019).

10 Orlhac, F. *et al.* A Guide to ComBat Harmonization of Imaging Biomarkers in Multicenter Studies. *J Nucl Med* **63**, 172-179, doi:10.2967/jnumed.121.262464 (2022).

11 Uher, R. *et al.* Measuring depression: comparison and integration of three scales in the GENDEP study. *Psychol Med* **38**, 289-300, doi:10.1017/s0033291707001730 (2008).

12 Klöbl, M. *et al.* Gender dysphoria and sexual euphoria – A Bayesian perspective on the influence of gender-affirming hormone therapy on sexual arousal. *medRxiv*, 2021.2011.2022.21266679, doi:10.1101/2021.11.22.21266679 (2021).

13 Folch-Fortuny, A., Arteaga, F. & Ferrer, A. Missing Data Imputation Toolbox for MATLAB. *Chemometrics and Intelligent Laboratory Systems* **154**, 93-100, doi:<https://doi.org/10.1016/j.chemolab.2016.03.019> (2016).

14 Lanzenberger, R. *et al.* Prediction of SSRI treatment response in major depression based on serotonin transporter interplay between median raphe nucleus and projection areas. *Neuroimage* **63**, 874-881, doi:10.1016/j.neuroimage.2012.07.023 (2012).

15 Imai, K., Keele, L., Tingley, D. & Yamamoto, T. in *Advances in Social Science Research Using R.* (ed Hrishikesh D. Vinod) 129-154 (Springer New York).

16 Kasper, S. *et al.* Receptor and Transporter Imaging Studies in Schizophrenia, Depression, Bulimia and Tourette's Disorder—Implications for Psychopharmacology. *The World Journal of Biological Psychiatry* **3**, 133-146, doi:10.3109/15622970209150614 (2002).

17 Praschak-Rieder, N. *et al.* Novel 5-HTTLPR allele associates with higher serotonin transporter binding in putamen: a [(11)C] DASB positron emission tomography study. *Biol Psychiatry* **62**, 327-331, doi:10.1016/j.biopsych.2006.09.022 (2007).

18 Praschak-Rieder, N., Willeit, M., Wilson, A. A., Houle, S. & Meyer, J. H. Seasonal variation in human brain serotonin transporter binding. *Arch Gen Psychiatry* **65**, 1072-1078, doi:10.1001/archpsyc.65.9.1072 (2008).

19 Harmer, C. J., Mackay, C. E., Reid, C. B., Cowen, P. J. & Goodwin, G. M. Antidepressant drug treatment modifies the neural processing of nonconscious threat cues. *Biol Psychiatry* **59**, 816-820, doi:10.1016/j.biopsych.2005.10.015 (2006).

20 Godlewska, B. R., Browning, M., Norbury, R., Cowen, P. J. & Harmer, C. J. Early changes in emotional processing as a marker of clinical response to SSRI treatment in depression. *Transl Psychiatry* **6**, e957, doi:10.1038/tp.2016.130 (2016).

21 Piel, J. H. *et al.* The effect of 5-HTTLPR and a serotonergic multi-marker score on amygdala, prefrontal and anterior cingulate cortex reactivity and habituation in a large, healthy fMRI cohort. *European Neuropsychopharmacology* **28**, 415-427, doi:<https://doi.org/10.1016/j.euroneuro.2017.12.014> (2018).

22 Harmer, C. J. & Cowen, P. J. 'It's the way that you look at it'--a cognitive neuropsychological account of SSRI action in depression. *Philos Trans R Soc Lond B Biol Sci* **368**, 20120407, doi:10.1098/rstb.2012.0407 (2013).

23 Wright, C. I. *et al.* Differential prefrontal cortex and amygdala habituation to repeatedly presented emotional stimuli. *Neuroreport* **12**, 379-383, doi:10.1097/00001756-200102120-00039 (2001).
